# Tumorigenic WAP-T Mouse Mammary Carcinoma Cells: A Model for a Self-Reproducing Homeostatic Cancer Cell System

**DOI:** 10.1371/journal.pone.0012103

**Published:** 2010-08-11

**Authors:** Florian Wegwitz, Mark-Andreas Kluth, Claudia Mänz, Benjamin Otto, Katharina Gruner, Christina Heinlein, Marion Kühl, Gabriele Warnecke, Udo Schumacher, Wolfgang Deppert, Genrich V. Tolstonog

**Affiliations:** 1 Department of Tumor Virology, Heinrich-Pette-Institute for Experimental Virology and Immunology, Hamburg, Germany; 2 Department of Clinical Chemistry/Central Laboratories, University Medical Center Hamburg-Eppendorf, Hamburg, Germany; 3 Department of Anatomy II: Experimental Morphology, University Medical Center Hamburg-Eppendorf, Hamburg, Germany; City of Hope National Medical Center, United States of America

## Abstract

**Background:**

In analogy to normal stem cell differentiation, the current cancer stem cell (CSC) model presumes a hierarchical organization and an irreversible differentiation in tumor tissue. Accordingly, CSCs should comprise only a small subset of the tumor cells, which feeds tumor growth. However, some recent findings raised doubts on the general applicability of the CSC model and asked for its refinement.

**Methodology/Principal Findings:**

In this study we analyzed the CSC properties of mammary carcinoma cells derived from transgenic (WAP-T) mice. We established a highly tumorigenic WAP-T cell line (G-2 cells) that displays stem-like traits. G-2 cells, as well as their clonal derivates, are closely related to primary tumors regarding histology and gene expression profiles, and reflect heterogeneity regarding their differentiation states. G-2 cultures comprise cell populations in distinct differentiation states identified by co-expression of cytoskeletal proteins (cytokeratins and vimentin), a combination of cell surface markers and a set of transcription factors. Cellular subsets sorted according to expression of CD24a, CD49f, CD61, Epcam, Sca1, and Thy1 cell surface proteins, or metabolic markers (e.g. ALDH activity) are competent to reconstitute the initial cellular composition. Repopulation efficiency greatly varies between individual subsets and is influenced by interactions with the respective complementary G-2 cellular subset. The balance between differentiation states is regulated in part by the transcription factor Sox10, as depletion of Sox10 led to up-regulation of Twist2 and increased the proportion of Thy1-expressing cells representing cells in a self-renewable, reversible, quasi-mesenchymal differentiation state.

**Conclusions/Significance:**

G-2 cells constitute a self-reproducing cancer cell system, maintained by bi- and unidirectional conversion of complementary cellular subsets. Our work contributes to the current controversial discussion on the existence and nature of CSC and provides a basis for the incorporation of alternative hypotheses into the CSC model.

## Introduction

The definition by Rollin Hotchkiss of living matter “as the repetitive production of ordered heterogeneity” is applicable to normal as well as to tumor tissue [1]. The cellular heterogeneity observed in many solid tumors at the functional and structural level is reminiscent to the complex cellular organization of the respective normal tissues. This similarity of tumor to normal tissue legitimizes the formal application of principles and concepts in developmental biology to cancer research. The model of cancer stem cells (CSCs) [2], [3] describes a tumor as a hierarchically organized system of stem-like cells and their differentiated progeny. As postulated by the CSC model, a small subset of cells drives tumor growth and is responsible for tumor relapse after an apparently successful therapy. These tumor cells, referred to as CSCs, tumor-initiating or tumorigenic cells, are distinguished by a combination of operationally defined common or unique cell surface associated markers and the ability to establish the disease in appropriate recipient mice [4]. In contrast to the stochastic model of clonal evolution, which ascribes tumor cell heterogeneity to genetic differences in the tumor cell pool [5], the CSC model postulates that epigenetic rather than genetic differences distinguish tumorigenic from non-tumorigenic cells, thereby providing a basis for the hierarchical relationships within the tumor cell population [6].

Recent findings that tumorigenic cells can comprise a significant fraction of the tumor mass [7] question the strictly hierarchical organization of the tumor tissue [8], and rather argue for “phenotypic plasticity” of tumor cells [9], maintained by homeostatic mechanisms [10]. Hence, CSCs do not exist as a unique population defined by discrete molecular properties, but rather together with their differentiated progeny constitute a self-reproducing “stem cell system” where the cellular composition is regulated by interconversion of various differentiation states [9]. Tumors of epithelial origin (carcinomas) usually display high histological heterogeneity reflecting various differentiation states of individual cells. Based on three phenotypic criteria - cell polarization, cell cohesiveness and expression pattern of cytoplasmic intermediate filament (cIF) proteins - it has been suggested to define four phenotypes, ranging from purely epithelial to entirely mesenchymal [11]. Accordingly, the differentiation state of individual cells in carcinomas corresponds to an epithelial, a mesenchymal and an intermediate phenotype. These differentiation states can be further subdivided into stable and transitory subtypes, which altogether are assembled into a dynamic “ecosystem”. The process termed epithelial-mesenchymal transition (EMT) and its counterpart, termed mesenchymal-epithelial transition (MET) [12], [13], describe the conversion of opposite differentiation states. These transitions have been recently linked to cell stemness by the observation that induction of EMT in human breast epithelial cell culture models creates a subset of cells highly enriched in CSCs [14], [15]. The model emerging from these studies proposes that in carcinomas EMT and MET account for the generation of a subset of cells which are in balance with the tumor epithelial compartment and are able to regenerate the whole tumor cell population [9].

Transgenic and knockout mice provide syngeneic (or congenic) models for CSC research, as they allow to establish cancer diseases in immune-competent animals that mimic the corresponding human situation, and are a source for cell lines enabling studies of CSC properties. However, the suitability of mouse models is often restricted by the fact that the effects of expression of an oncogene, or loss of a tumor suppressor, are exerted already at the embryonic stage and during tissue development, while in the vast majority of human cancers genetic alterations leading to cancer will occur in cells of adult tissues. WAP-T transgenic mice [16]–[19] have proven to be a useful model for the analysis of oncogene-induced mammary carcinogenesis in adult mice. In female WAP-T mice activation of the transgene, the simian virus 40 (SV40) early gene region flanked by an ∼1.4 kb upstream region of the gene coding for the mouse whey acidic protein (WAP) [20], is initiated during late pregnancy in mammary epithelial (ME) cells concordant with the endogenous *Wap* gene [21]. Expression of SV40 early genes coding for large T-antigen (LT) and small t-antigen (st) drives mammary carcinogenesis by mimicking a variety of genetic alterations commonly seen in human breast carcinomas, like abrogation of the pRB-controlled G1-checkpoint [22], and inactivation of the tumor suppressor p53 [23]. As a consequence of SV40 early gene expression, parous WAP-T mice develop multiple alveolar lesions – multifocal intraepithelial neoplasia (MIN). Some of these focal lesions further progress to invasive, but rarely metastatic mammary carcinomas [19]. Morphologically, the tumors developing in WAP-T mice are adenocarcinomas, ranging from a well to a poorly differentiated phenotype [16]. The relevance of this model is emphasized by the close similarity in histology of the mouse tumors with corresponding human tumors [19].

In this study we asked whether WAP-T tumors are better described by the classical CSC model or by alternative hypotheses. We found that tumorigenic cells are relatively frequent in WAP-T tumors (up to 1/10) and are able to recapitulate the phenotype of their respective primary tumors after orthotopic transplantation into syngeneic mice. To study their tumorigenic properties in more detail, we established from a WAP-T tumor a cell line (G-2 cells). G-2 cells with high efficiency form tumors in syngeneic mice which by gene expression and phenotypic analyses are closely related to primary tumors. G-2 cell cultures are characterized by a basal/luminal gene expression signature, heterogeneity of differentiation states and the presence of complementary cellular subsets that can be separated according to differences in expression of certain stemness-related cell surface markers. We show that stringently FACS-separated subsets of G-2 cells are competent to reconstitute the initial cellular composition of the cell culture when individually cultured. Our data argue for a self-reproducing homeostatic “cancer cell system”, where the balance relies on interconversion of the complementary cellular subsets, their interactions and transcriptional competence. In support of the EMT-CSC model [9], we identified in the G-2 culture a self-renewing population of cells characterized by expression of Thy1 and displaying spontaneous reversibility of a quasi-mesenchymal differentiation state.

## Results

### WAP-T tumors contain a high proportion of tumorigenic cells

**Figure 1:**
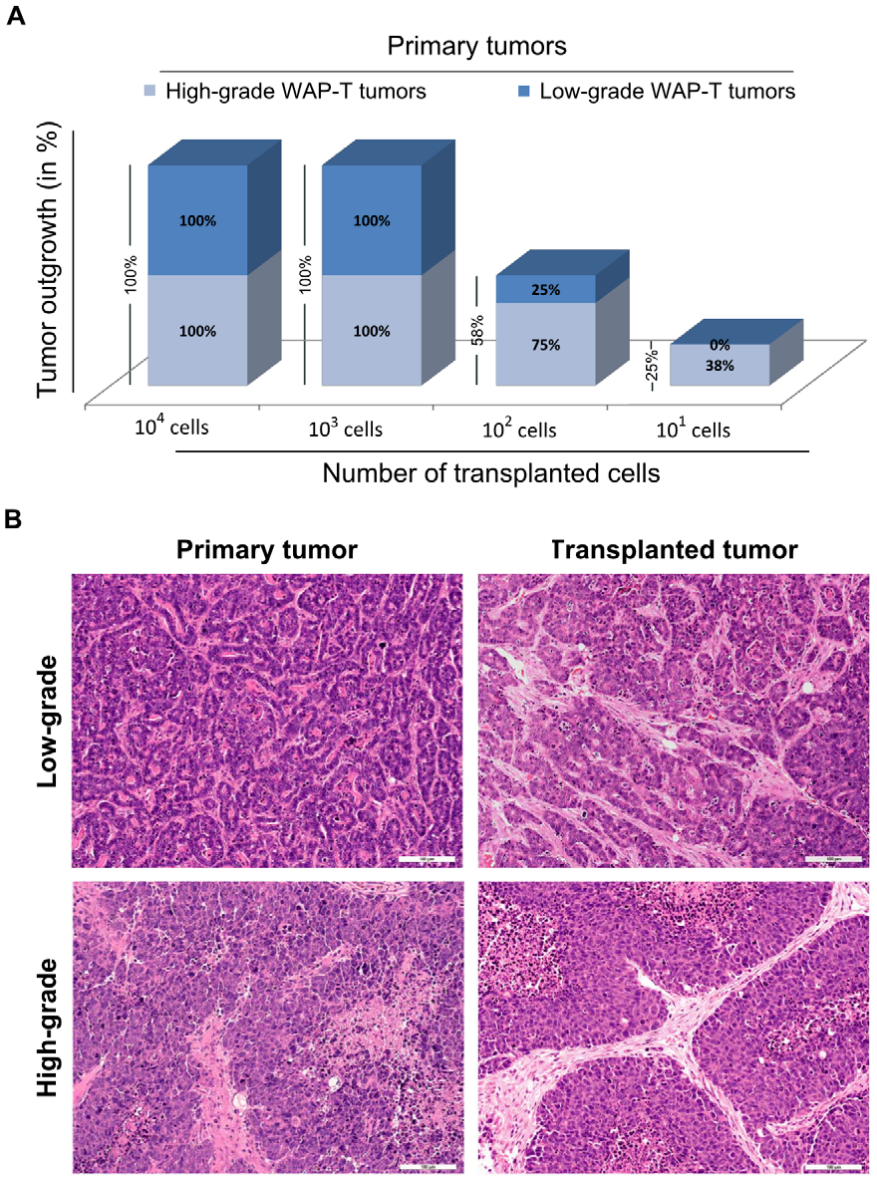
Tumorigenic property of WAP-T tumor cells. (**A**) Cells from high-grade WAP-T tumors are more tumorigenic than cells from low-grade tumors. Serially diluted (10^1^, 10^2^, 10^3^, 10^4^) freshly isolated WAP-T tumor cells were injected into the left abdominal mammary gland as described in **Materialsand Methods**. (**B**) H&E staining of low-grade and high-grade, respectively, WAP-T primary and their corresponding transplanted tumor. The transplanted tumors grew after injection of 10^4^ or 10^2^ cells, respectively, from low-grade and high-grade tumors. Scale bar: 100 µm.

A decisive criterion for CSCs is their ability to initiate tumor growth after transplantation into appropriate recipient mice and to recapitulate the phenotype of the original tumor. Orthotopic transplantation of serially diluted WAP-T tumor cells revealed that as low as 10^2^ cells from well to moderately differentiated (low-grade) tumors, and as low as 10^1^ cells from poorly differentiated (high-grade) tumors were able to induce mammary carcinomas in syngeneic mice (Figure 1A). Transplanted tumors usually reflected the phenotype of the parental tumors (Figure 1B). However, transplantation of cells from a low-grade tumor sometimes also gave rise to high-grade tumors. As pauci-clonality of WAP-T tumors has been occasionally observed [17], the outgrowth of cells from a high-grade tumor cell pool cannot be excluded.

### Characterization of G-2 cells and their clonal derivatives

To avoid the complications associated with the analysis of primary tumor cells, we established a cell line from a WAP-T tumor (G-2 cells) that would allow analysis of mammary tumor initiating and stem cell properties under *in vitro* and *in vivo* settings (see Materials and Methods for details).

#### a) *In vitro*

**Figure 2:**
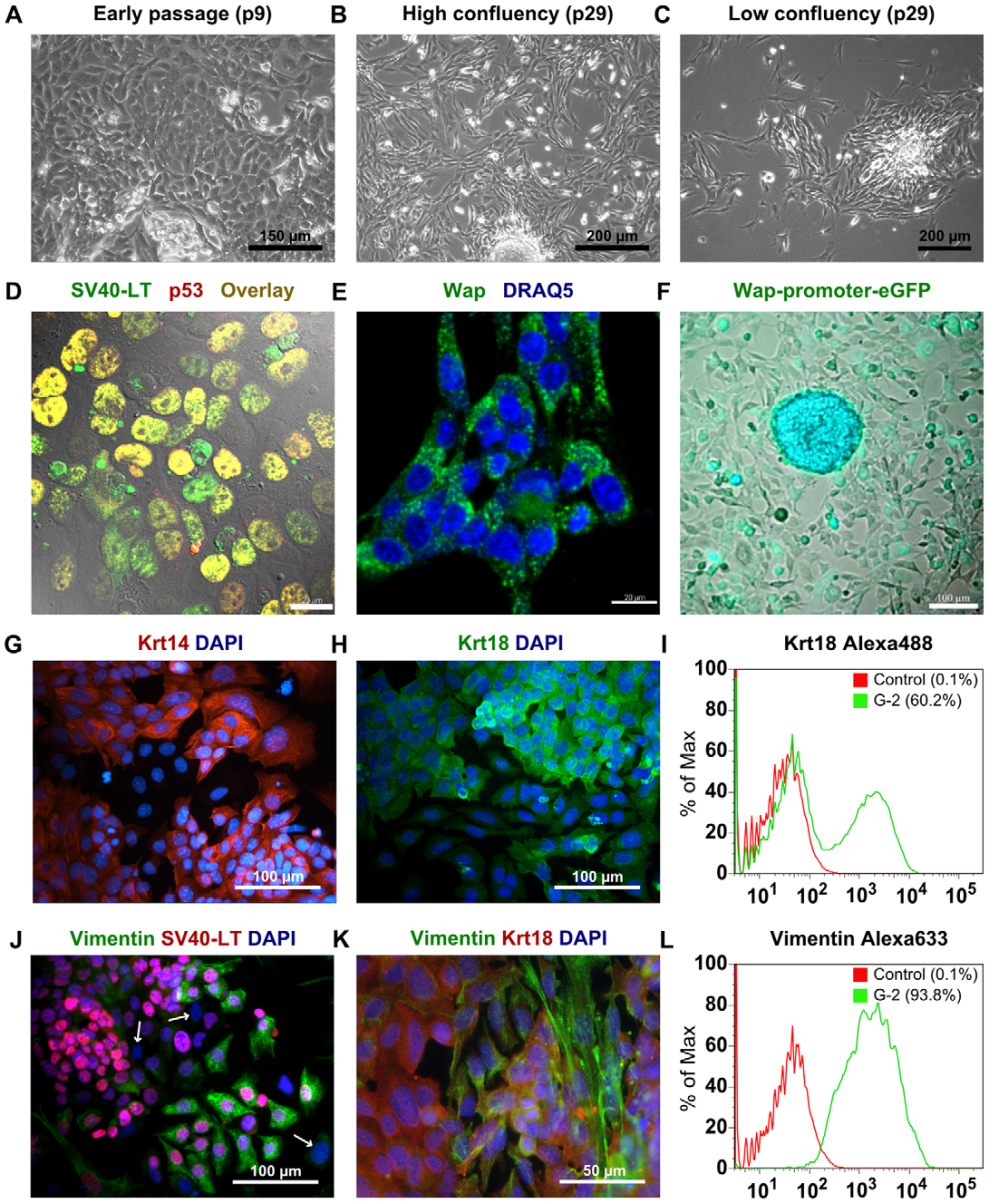
Characterization of G-2 cells in cell culture. (**A–C**) Phase-contrast images of G-2 cells in an early (p9) and a later (p29) passage. (**D**) Confocal image of G-2 cells stained with anti-SV40-LT (green) and anti-p53 (red) antibodies. Images were merged with a differential interference contrast (DIC) micrograph. (**E**) Confocal image of G-2 cells stained with anti-Wap (green) antibody. Nuclei were visualized by DRAQ5 staining (blue). (**F**) Live-cell fluorescence image of G-2 cells after lentiviral transduction with an eGFP reporter construct under control of the *Wap*-promoter. FACS-enriched eGFP^+^-cells were kept in culture for 2 weeks. (**G** and **H**) Keratin 14 (red) and keratin 18 (green) immunostaining of cultured G-2 cells. Nuclei were visualized by DAPI staining (blue). (**I**) FACS-based quantitation of keratin 18 expression in G-2 cells at passage 20. (**J** and **K**) Vimentin (green) and SV40-LT (red)/keratin 18 (red) co-staining of cultured G-2 cells at passage 10. Arrows mark G-2 cells without detectable SV40-LT expression. Nuclei were visualized by DAPI staining (blue). (**L**) FACS-based quantitation of vimentin expression in G-2 cells at passage 20. Scale bars: **A**: 150 µm; **B** and **C**: 200µm; **D** and **E**: 20 µm; **F**, **G**, **H** and **J**: 100 µm; **K**: 50µm.

Starting from the first passages G-2 cell cultures exhibited an inhomogeneous growth pattern, featured by tightly packed colonies embedded into cobblestone-like areas (Figure 2A). In subsequent passages G-2 cells preserved the ability to form multiple cell clusters and three-dimensionally expanding colonies, but acquired a more fibroblastic-like morphology (Figure 2B, C). G-2 cells exhibit stable, though heterogeneous expression of SV40-LT, which in complex with the endogenous wild-type p53 accumulated in the nuclei of the majority of cells (Figure 2D), thereby reflecting SV40-LT expression *in vivo*. Expression of SV40-LT correlates with endogenous *Wap* gene activity (Figure 2E), indicating that regulators responsible for transcription of the *Wap* gene are constitutively active in G-2 cells. In support, lentiviral transduction of G-2 cells with a GFP reporter construct under control of a ∼1.4 kb *Wap* promoter fragment showed that even after 2 weeks in culture a large population of the FACS-enriched eGFP^+^-cells remained eGFP-positive (Figure 2F). These cells occasionally formed dense foci of highly eGFP expressing cells.

Since constitutive *Wap* gene activity has been linked to committed bipotent alveolar progenitors and CD61^+^ luminal-restricted progenitors, which are presumptive targets of oncogene activity [24], we performed a cell lineage analysis by immunofluorescence (IF) staining for the luminal epithelial cell marker keratin 18 (Krt18), the basal/myoepithelial cell markers keratin 5 (Krt5) and keratin 14 (Krt14). In early passages the majority of the G-2 cells expressed both Krt14 (Figure 2G) and Krt18 intermediate filament proteins (Figure 2H); however the usual co-polymerization partner of Krt14, the Krt5 protein, was not detectable by a specific antibody (data not shown). In subsequent passages slight variations in the individual expression levels of Krt14 and Krt18 (data not shown) and a significant fluctuation in the number of cytokeratin-expressing cells ranging from 40 to 70% were observed. Figure 2I shows as an example the FACS-based quantitation of Krt18 expression in G-2 cells at passage 20, indicating transition towards a mesenchymal differentiation state. Therefore, expression of the intermediate filament protein vimentin was analyzed as a widely-used marker of mesenchymal cells and carcinoma cells undergoing transition between epithelial and mesenchymal differentiation states [25], [26]. Independent of passage number nearly all G-2 cells express vimentin, whereby the intensity of vimentin expression ranges from a diffuse cytoplasmic distribution and faint filamentous structures to an abundant filamentous network (Figure 2J–K show vimentin/SV40-LT and vimentin/Krt18 co-staining at passage 10). Figure 2L shows as an example the FACS-based quantitation of vimentin expression in G-2 cells at passage 20. SV40-LT expression was detected almost always also in cells strongly expressing vimentin (Figure 2J). However, few cells lacking SV40-LT were always present in G-2 cultures (Figure 2J, labeled by arrows). In summary, by immunostaining analysis we observed firstly that the majority of G-2 cells are distinguished by a differentiation state characterized by co-expression of vimentin and cytokeratins, secondly that the number of cells devoid of cytokeratins fluctuates between passages, and thirdly that a minor population of cells expressing cytokeratins but totally lacking vimentin is always present.

Such heterogeneity in differentiation states may reflect a multiclonal origin of the G-2 cell culture, as this culture was derived from a whole tumor. To address this possibility, G-2 cells were cloned in soft agar, and ten colonies were expanded into stable cell lines. As visualized by IF-staining, the G-2 cell pattern of cytokeratin expression was reproduced in G-2 cell derived clones (Figure S1A–B; shown as example for clones G-2C9 and G-2C11), although according to qPCR analysis the relative levels of *Krt14* and *Krt18* gene expression varied markedly between clones (Figure S1C–D). Also in the secondary clones, derived by agar cloning from G-2C9 and G-2C11 clones, a 2–3 fold variation in the transcription of *Krt14* and *Krt18* genes could be demonstrated (Figure S1E–F). Similar to the parental culture, heterogeneous expression of vimentin was observed in G-2 cell clones (Figure S1G). Therefore, we conclude that the presence of cell populations in epithelial, mesenchymal and intermediate differentiation states is an inherent property of G-2 cultures.

#### b) *In vivo*

**Figure 3:**
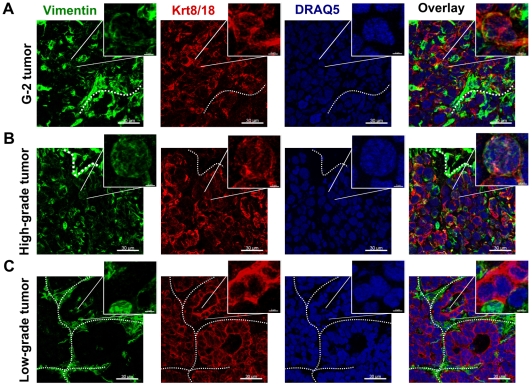
Expression of the intermediate filament proteins in G-2 and WAP-T tumors. (**A–C**) Representative confocal images of tumor-cryosections of a G-2 cell-derived tumor (**A**), and a high-grade (**B**) and a low-grade (**C**) endogenous WAP-T tumor stained with anti-vimentin (green) and anti-keratin 8/18 (red) antibodies. The nuclei were stained with DRAQ5 (blue). The power insets are used to display co-expression of vimentin and keratin 8/18 in G-2 and high-grade WAP-T tumors. The white dashed lines mark stromal structures. The confocal 3D-stacks were deconvoluted using Huygens Essential software and reconstructed with the Imaris software. Scale bar: main picture: 30 µm; magnification: 3 µm.

**Figure 4:**
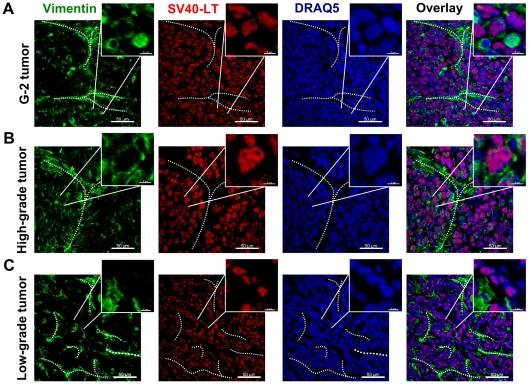
Expression of the intermediate filament protein vimentin and the SV40-LT transgene in G-2 and WAP-T tumors. (**A–C**) Representative confocal images of tumor-cryosections of a G-2 cell-derived tumor (**A**), a high-grade (**B**) and a low-grade (**C**) endogenous WAP-T tumor stained with anti-vimentin (green) and anti-SV40-LT (red) antibodies. The nuclei were stained with DRAQ5 (blue). The power insets are used to display co-expression of vimentin and SV40-LT in G-2 and high-grade WAP-T tumors. The white dashed lines mark stromal structures. The confocal 3D-stacks were deconvoluted using Huygens Essential software and reconstructed with the Imaris software. Scale bar: main picture: 50 µm; magnification: 6 µm.

**Figure 5:**
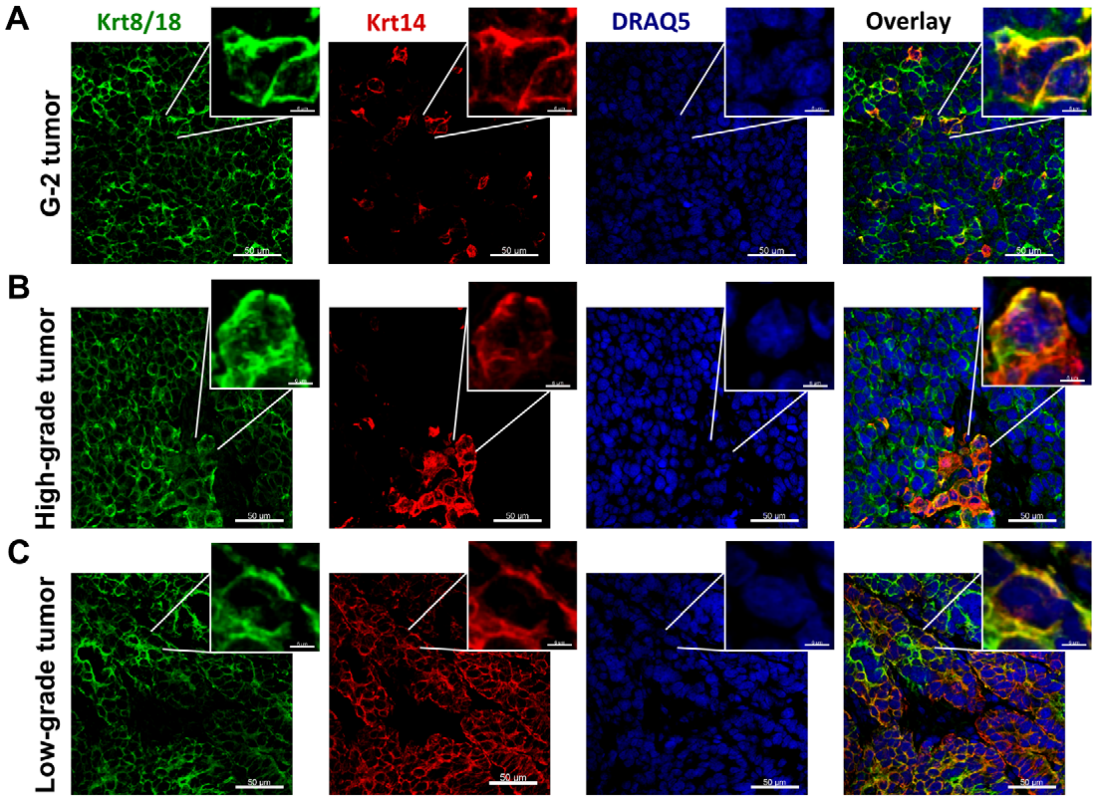
Expression of the cytokeratins in G-2 and WAP-T tumors. (**A–C**) Representative confocal images of tumor-cryosections of a G-2 cell-derived tumor (**A**), a high-grade (**B**) and a low-grade (**C**) endogenous WAP-T tumor stained with anti-keratin 8/18 (green) and anti-keratin 14 (red) antibodies. The nuclei were stained with DRAQ5 (blue). The power insets are used to display co-expression of keratin 8/18 and keratin 14 in G-2, high-grade and low-grade WAP-T tumors. The confocal 3D-stacks were deconvoluted using Huygens Essential software and reconstructed with the Imaris software. Scale bar: main picture: 50 µm; magnification: 6 µm.

We tested the tumor initiating potential of G-2 cells by orthotopic transplantation into the left abdominal mammary gland of nulliparous WAP-T recipient mice. 10^6^ G-2 cells rapidly formed palpable tumors (high-grade adenocarcinomas) in all recipient mice (Table 1). The fewer cells we transplanted, the longer was the lag period and the higher its variation (Table 1). Even from 10 injected G-2 cells in 10 out of 12 recipient mice tumor outgrowth was detected (Table 1), indicating a high frequency of tumorigenic cells in the G-2 culture. Immunostaining analysis of vimentin and Krt8/18 expression in G-2 cell derived tumors (Figure 3A) revealed their close resemblance to poorly differentiated (grade G3) WAP-T tumors (Figure 3B). While in low-grade WAP-T tumors vimentin staining was mostly limited to septa and the stromal compartment (Figure 3C), variable expression of vimentin was also detectable in the epithelial compartment (represented by Krt8/18) in high-grade WAP-T tumors (Figure 3B), indicating an intermediate differentiation state of tumor cells in G-2 derived and high-grade WAP-T tumors and an epithelial differentiation state of tumor cells in low-grade WAP-T tumors. Co-staining for vimentin and SV40-LT (expression of SV40-LT is limited to tumor cells) allows to distinguish between stromal mesenchymal cells recruited into the tumors and tumor cells expressing a mesenchymal or an intermediate phenotype. In transplanted G-2 tumors (Figure 4A) and high-grade WAP-T tumors (Figure 4B) we observed in addition to the expected expression of vimentin in the stromal compartment also the co-expression of vimentin and SV40-LT in individual tumor cells. In contrast, in low-grade WAP-T tumors (Figure 4C) vimentin and SV40-LT expression is restricted to stromal and tumor epithelial compartments, respectively. Tumors grown from transplanted G-2 cells (Figure 5A) recapitulated the distinctive features of high-grade WAP-T tumors (Figure 5B), namely a moderate proportion of cells co-expressing Krt14 and Krt8/18. In contrast, in low-grade WAP-T tumors Krt8/Krt18 and Krt14 are frequently co-expressed (Figure 5C).

**Table 1:** Tumorigenic properties of G-2 cells*.

Number of injected cells	Tumors/injections	Outgrowth latency (Mean of days ± SEM)
10^6^	6/6	7.3±0.4
10^3^	6/6	16.3±6.3
10^2^	5/6	23.8±10.9
10^1^	10/12	44.8±24.2

*10^6^, 10^3^, 10^2^ and 10^1^ G-2 cells were resuspended in 20 µl BD Matrigel Matrix and transplanted into the left abdominal mammary gland of virgin WAP-T-NP8 recipient mice. The animals were palpated twice a week for tumor outgrowth.

In an additional experiment, 10^6^ G-2 cells were transplanted into fad pads of two non-transgenic BALB/c mice. In both mice large, solid tumors grew after 4–6 weeks. Interestingly, the tumor cells were largely devoid of epithelial markers, Epcam (Figure S2A) and cytokeratins (Figure S2B, D), but strongly expressed vimentin (Figure S2B, C), indicating that in non-transgenic mice transition into the mesenchymal state is favored, possibly as a consequence of an interaction with the host immune system. As the tumor cells continue to express WAP-promoter driven SV40-LT (Figure S2C), it is likely that mesenchymal differentiation in these cells was incomplete.

#### c) Gene expression profiling

**Figure 6:**
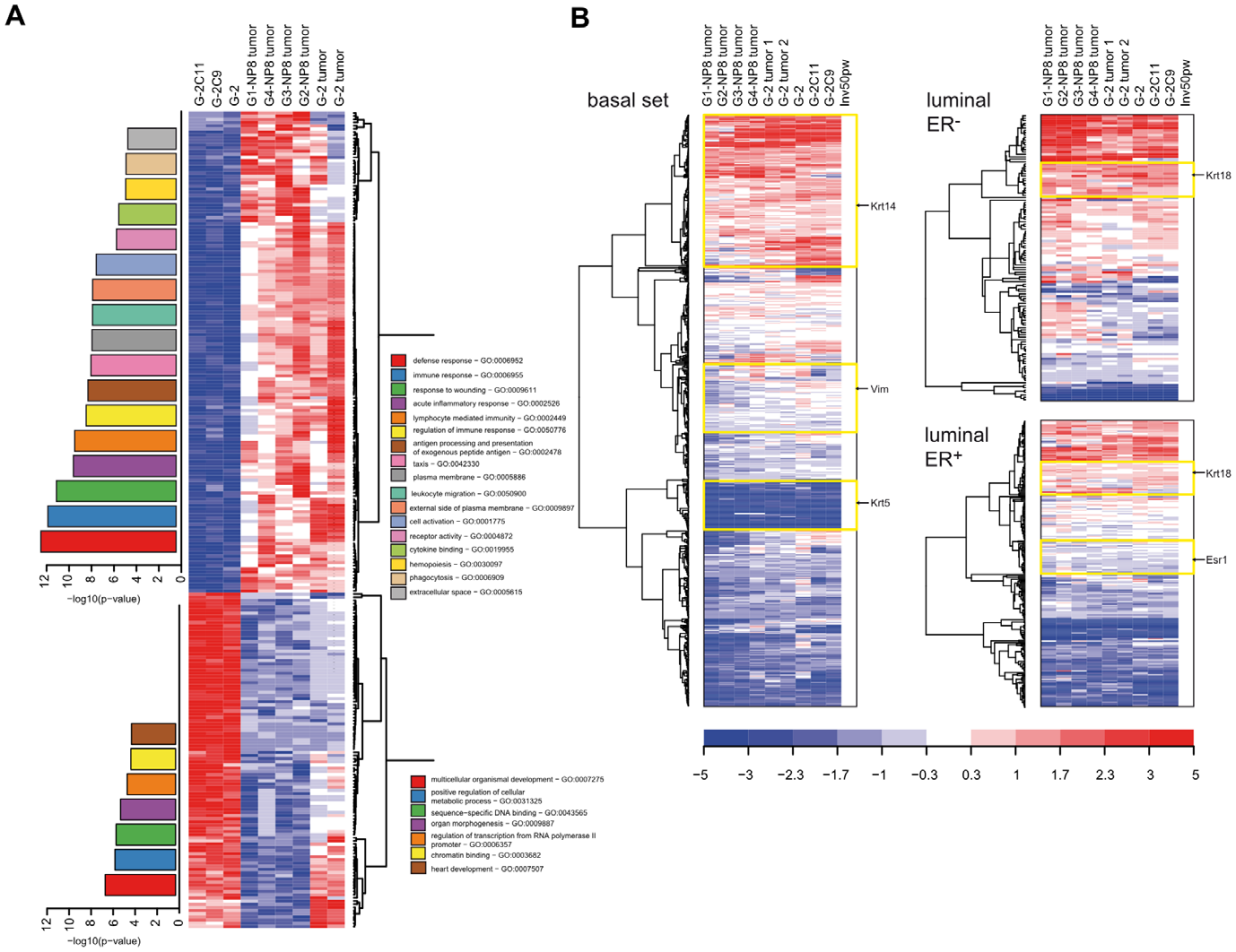
Gene expression profiling of G-2 cells and tumor samples. (**A**) 250 genes differentially expressed in cultured G-2 cells and endogenous WAP-T or G-2 cell derived tumors (Table S1) were used to generate a heat map. Enriched GO categories are shown as bar diagrams corresponding to higher or lower expressed gene clusters in the respective sample group. Color-coding and the height of a bar represents the statistical significance (-log_10_(p-value)) of the observed enrichment of the respective GO categories. (**B**) Genes characteristic for luminal-ER^+^, luminal-ER^−^, and basal/myoepithelial cells were used to generate heat maps. Gene expression data obtained for cell culture (G-2, G-2C9 and G-2C11 cells) and tumor samples (two G-2 transplanted tumors and four WAP-T-NP8 tumors representing four histological grades) were used for this analysis. Gene expression intensities of a mammary gland of a parous BALB/c mouse (50 days post weaning) were used as a reference. Prominent gene clusters (*Krt14*, *Vim*, *Krt5*, *Krt18*, and *Esr1*) are highlighted by yellow boxes. The expression values are color coded: red – high expression, blue – low expression.

The histomorphological similarity between G-2 cell transplanted and WAP-T tumors indicates that these tumors might also be closely related at the molecular level. Based on the comparable expression of cIF proteins, we also expected a close relationship between G-2 cells in culture and in G-2 tumors. To test these assumptions, we performed microarray expression profiling. Total RNA from G-2, G-2C9 and G-2C11 cells, from two G-2 transplanted tumors, as well as from four WAP-T-NP8 tumors representing four histological grades (G1–G4) was analyzed on an Affymetrix microarray platform (MOE430 2.0). Applying as significance criteria the corr. P-value (Benjamini-Hochberg) < = 0.05, and a fold change-cutoff 3, we identified 250 genes that were differentially expressed between cell culture and tumor samples (Table S1). Tightening the statistical criteria to a corr. P-value < = 0.01, only 24 genes satisfied these strict criteria. The 250 differentially expressed genes were further analyzed by the EXPANDER program in order to identify over-represented GO (gene ontology) categories and TF (transcription factor) binding sites in their *cis*-regulatory regions [27]. The GO-enrichment analysis was combined with hierarchical clustering, and the results are presented in the heat map shown in Figure 6A. A significant number of differentially expressed genes falls into the category of immune defense genes, reflecting the fact that tumors contain a certain contingent of immune cells, which is missing in cell culture. The enrichment of genes related to immune processes correlates well with the over-representation of binding sites for Elf1, a transcription factor highly expressed in lymphoid cells [28], in the promoter regions of the differentially expressed genes (P-value <0.001). On the other hand, the cell culture samples are distinguished by a higher expression of genes associated with transcriptional regulation, e.g. *Foxa2*, *Foxm1*, *Gata6*, *Hoxb2*, *Jmjd2c*, *Ppargc1a*, and *Tle1*, and developmental processes, e.g. *Bmp4* which is involved in the differentiation of mesenchymal cells. This observation can be explained by adaptation of the transcriptional network and of signaling pathways to cell culture conditions. Taken together, the gene expression analysis demonstrated that G-2 cells and tumors display more similarities than differences in their gene expression program; the differences are mainly related to their different biological context.

We reckoned that gene expression analysis should help to locate G-2 cells within the mammary epithelial cell hierarchy. Using a list of genes characteristic of luminal-ER^+^, luminal-ER^−^, and basal/myoepithelial cells [29] (Table S1), and the gene expression profile of the mammary gland of a parous BALB/c mouse (50 days post weaning) as a reference, hierarchical cluster analysis again revealed similarity between cell culture and tumor samples (Figure 6B). Furthermore, a complex transcriptional program comprising many genes from all three lineages became obvious. Notably, *Esr1* (coding for estrogen receptor alpha) is inside a cluster of down-regulated “luminal-ER^+^” genes, *Vim* (coding for vimentin) in the cluster of non-regulated genes, *Krt14* is located in the cluster of up-regulated “basal” genes, whereas *Krt5* together with a number of other genes forms a cluster of down-regulated “basal” genes, consistent with the absence of Krt5 expression in G-2 cell cultures and the rare occurrence of Krt5-postive cells in WAP-T tumors (data not shown). Based on this analysis, we conclude that the G-2 cell transcriptome likely is related to cells committed to luminal-ER^−^ differentiation. These cells could be progenitors of luminal-ER^−^ cells, which during commitment lost the expression of prominent markers, like *Esr1* and *Krt5* – genes functionally linked to luminal-ER^+^ and basal cell lineages, and became immortalized by SV40 proteins. However, final clarification of their identity requires further studies.

### Stemness of G-2 cells and their clonal derivatives

The high tumorigenic potential of G-2 cells prompted us to study their CSC properties in more details. We performed a number of standard assays to measure expression of a set of cell surface markers, self-renewal, and generation of phenotypically different progeny (collectively termed repopulation activity), activity of aldehyde dehydrogenases, and colony forming potential.

#### a) Cell surface markers

**Figure 7:**
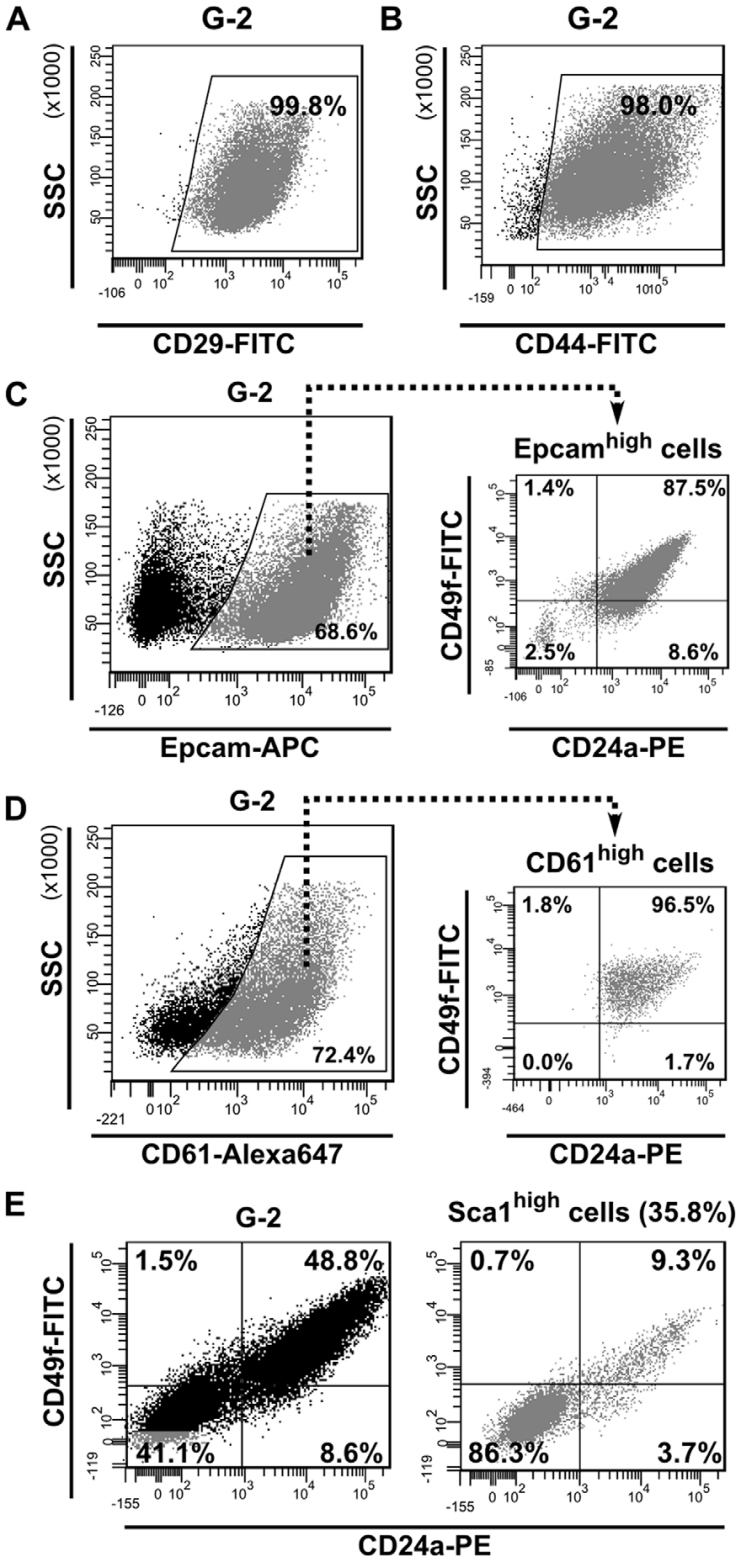
Expression of stemness-related cell surface markers in G-2 cells. (**A, B**) Representative FACS dot plots showing the expression of CD29 (**A**) and CD44 (**B**) in G-2 cultured cells. (**C**) Representative FACS dot plots showing the expression of Epcam (**C**, left) in cultured G-2 cells and the distribution of CD24a and CD49f (**C**, right) within the Epcam^high^ cell population. (**D**) Representative FACS dot plots showing the expression of CD61 (**D**, left) in cultured G-2 cells and the distribution of CD24a and CD49f (**C**, right) within the CD61^high^ cell population. (**E**) Representative FACS dot plots showing the expression of CD24a and CD49f in cultured G-2 cells (**E**, left) and within the Sca1^high^ (**E**, right) cell population.

Expression of certain cell surface associated proteins, like integrins and GPI-anchored proteins, is diagnostic for normal mammary stem cells and breast cancer stem cells. By FACS we observed that nearly all G-2 cells express the stemness-related markers CD29 (integrin beta 1) [30] (Figure 7A) and CD44 (hyaluronate receptor) [31] (Figure 7B). A large fraction of G-2 cells is positive for Epcam (epithelial cell adhesion molecule) [31], [32] (Figure 7C, left), which is co-expressed with CD24a and CD49f (integrin alpha 6) proteins [30], [33]–[35] (Figure 7C, right). Another mammary progenitor-associated marker, CD61 (integrin beta 3) [36], was detected on a large fraction of G-2 cells (Figure 7D, left) and is also co-expressed with CD24a and CD49f (Figure 7D, right). Therefore, we collectively termed the fraction of G-2 cells co-expressing these cell membrane associated proteins as the CD24a^high^ subset. Variations ranging from ∼30% to ∼90% in the absolute number of cells in this subset were noted between parental G-2 cells and clones (data not shown). Next, we observed that the counterpart of the CD24a^high^ subset (Figure 7E, left) is characterized by expression of the stem cell marker Sca1 [37] (Figure 7E, right). Taken together, the G-2 culture comprises two major distinct subsets, CD24a^high^/Sca1^low^ and CD24a^low^/Sca1^high^. SV40 transgene is equally transcribed in both subsets, but the transcription of Krt14 and Krt18 genes is higher in CD24a^high^/Sca1^low^ cells (Figure S3A, B). We also observed by qPCR analysis that *Cd24a*, *Cd49f* and *Sca1* genes are transcribed in both subsets (Figure S3C).

**Figure 8:**
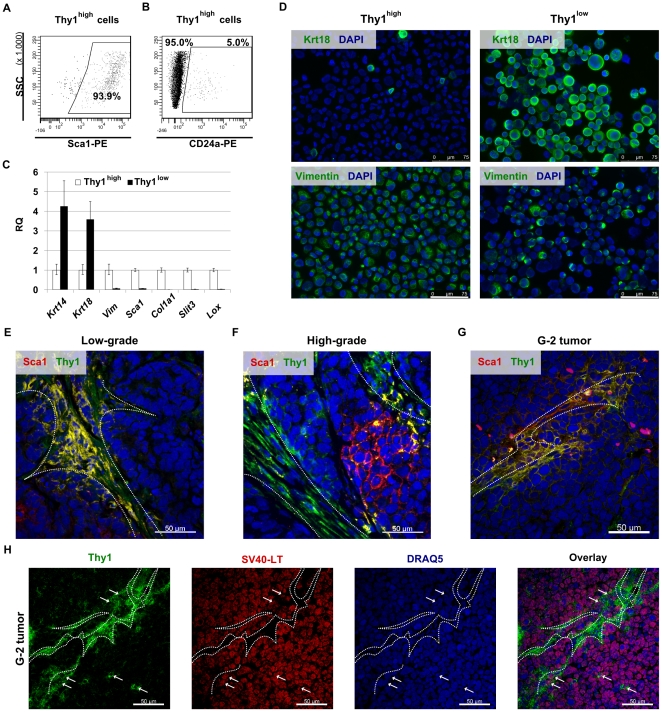
Properties of G-2 cell subsets differing in expression of Thy1. (**A, B**) Representative FACS dot plots showing the expression of Sca1 (**A**) and CD24a (**B**) in the Thy1^high^ population of cultured G-2 cells. (**C**) Relative quantitation of gene expression by real-time qPCR in the Thy1^high^ and Thy1^low^ subsets. Each assay was done in triplicate and raw RQ values were calculated by normalizing to the *Gapdh* gene. The Thy1^high^ subset was selected as calibrator. (**D**) Cytospin preparations of FACS-sorted Thy1^high^ and Thy1^low^ G-2 cells were stained for keratin 18 (**D**, upper panels) and vimentin (**D**, lower panels). (**E, F, G**) Representative confocal images of a low-grade (**E**) and a high-grade (**F**) WAP-T tumors as well as of a G-2 (**G**) tumor. Cryosections of tumor samples were stained with anti-Thy1 (green) and anti-Sca1 (red) antibodies. (**H**) Representative confocal image showing expression of Thy1 (green) and SV40-LT (red) in G-2 tumor. Individual color channels are shown. Arrows mark the cells co-expressing Thy1 and SV40-LT. Nuclei were stained with DAPI (**D**) or DRAQ5 (**E, F, G, H**). The white dashed lines mark stromal structures. Confocal 3D-stacks were deconvoluted using Huygens Essential software and reconstructed with the Imaris software. Scale bar: **D**: 75 µm; **E**, **F**, **G** and **H**: 50 µm.

Recently, a small subset of Thy1^+^/CD24^+^ cells purified from MMTV-*Wnt*-1 mammary carcinomas has been found to behave like CSCs [33]. In the G-2 culture, the cells distinguished by Thy1 expression represent an always variably sized population accounting for less than 15% (Figure S4A). In contrast to the report by Cho et al. [33], we observed that Thy1^high^ cells are mainly contained within the CD24a^low^/Sca1^high^ subset (Figure 8A); however a small fraction of cells co-expressed Thy1 and CD24a (Figure 8B). In adherently growing cells, membrane-associated expression of Thy1 coincides with that of Sca1 (Figure S4B, left), whereas Epcam-positive cells are negative for Thy1 (Figure S4B, right).

When we compared microarray data for two clones, G-2C9 and G-2C11, with parental G-2 cells as a reference, we observed that the list of genes weaker expressed in the subclones (FC>3; list of genes is in Table S1) includes the *Thy1* gene and genes coding for the transcription factor Twist2, which promotes metastatic spreading [38], the secreted protein Slit3, which is expressed in mammary gland [39] and fetal lung mesenchyme [40], the lysyl oxidase (Lox) involved in the crosslinking of collagens and the formation of a premetastatic niche [41], and several collagen-coding genes (*Col1a1*, *Col1a2*, *Col5a1*, *Col5a2*, *Col8a1* and *Col12a1*). We reasoned that transcription of these genes is linked to the Thy1^high^ phenotype. Indeed, the Thy1^high^ subset is distinguished by a nearly exclusive expression of *Col1a1*, *Slit3*, and *Lox*, and as expected from FACS analysis, by *Sca1* (Figure 8C). The transcription levels of *Cd24a* and *Cd49f* genes are low, but clearly detectable in the Thy1^high^ subset (Figure S4C). The data indicate that the Thy1^high^ subset represents a cellular subset in an apparent mesenchymal differentiation state, which, however, is not complete, as indicated by the transcription of the *Krt14* and *Krt18* genes (Figure 8C) in addition to the *Cd24a* and *Cd49f* genes. By immunostaining of cytospin preparations of FACS-sorted cells we were able to show that only a minor population of the Thy1^high^ cells is clearly positive for cytokeratins, as opposed to the prominent expression of vimentin in all Thy1^high^ cells (Figure 8D; Figure S4D). On the other hand, cytokeratins are detectable in nearly all Thy1^low^ cells, whereas (a generally reduced) positive vimentin immunostaining is limited to roughly two-thirds of the Thy1^low^ cells (Figure 8D; Figure S4D). The few Thy1^high^ cells expressing Krt18 may represent either contaminating cells, or cells which are in process of transition towards the Thy1^low^-state (see below). Notably, despite underrepresentation of cytokeratin-positive cells within the Thy1^high^ subset, the overall transcription of *Krt14* and *Krt18* genes is only 4-fold stronger in Thy1^low^ cells than in Thy1^high^ cells (Figure 8C).

To analyze Thy1 expression in WAP-T and G-2 tumors the samples were immunostained with a Thy1-specific antibody. In low-grade WAP-T tumors we noted that Thy1 as well as Sca1 expression is mostly restricted to the stromal compartment and to adjacent tumor cells (Figure 8E). In high-grade carcinomas a subset of cells adjacent to the stroma, demarked by the strong expression of Thy1, expresses either Thy1 or Sca1 or both markers (Figure 8F). Similarly, in tumors grown from G-2 transplanted cells the membranes of cells adjacent to Thy1-positive stromal cords are weakly stained with the Thy1 antibody (Figure 8G). The tumor origin of Thy1-expressing cells was further confirmed by co-staining with the SV40-LT specific antibody (Figure 8H), which we used to distinguish between tumor cells and cells provided by recipient mice.

#### b) Repopulation activity

**Figure 9:**
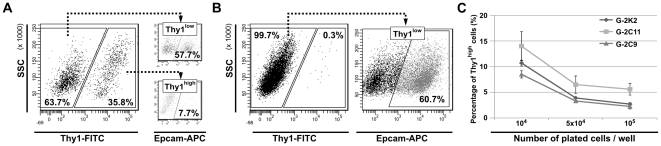
Repopulation activity of the Thy1^high/low^ G-2 cell subsets. (**A, B**) Repopulation activity of the Thy1^low^ and Thy1^high^ subsets. FACS-sorted Thy1^high^ (**A**) and Thy1^low^ (**B**) G-2 cells were cultured for 5 days in 6-well plates and re-analyzed by FACS for Thy1 expression (**A**, left; **B**, left). The distribution of Epcam within the respective Thy1^high^ and Thy1^low^ subsets was then determined by further FACS-analysis (**A**, **B**, right panels). (**C**) Cells of the G-2 derived clones K2, C9 and C11 were plated at low (1×10^4^), intermediate (5×10^4^) and high (1×10^5^) density in 6-well plates and analyzed 2 days later for Thy1 expression by FACS (n = 3). The representative FACS dot plots are shown.

Next, G-2 cells were separated according to Thy1 expression and after 5 days in culture re-analyzed by FACS. The Thy1^high^ subset repopulated both Thy1 subsets (Figure 9A, left), thereby creating a cell population that was nearly identical to the initial G-2 culture with regard to expression of Epcam (Figure 9A, right). In contrast, almost all Thy1^low^ cells remained in the Thy1^low^ state (Figure 9B, left), but generated a population of cells consisting of Epcam^high^ and Epcam^low^ subsets (Figure 9B, right). The observed limited repopulation activity of Thy1^low^ cells indicates that regeneration of the Thy1^high^ subset either requires longer cultivation or depends on culture conditions. The latter possibility is supported by a significant decrease of the Thy1^high^ subset as a function of increasing cell density (Figure 9C). Accordingly, the lower the cell density, the more Thy1^high^ cells were present in the cell culture. This could explain the significant variations in the proportion of Thy1^high^ cells observed between passages (data not shown) and within G-2 clones (Figure S4A).

**Figure 10:**
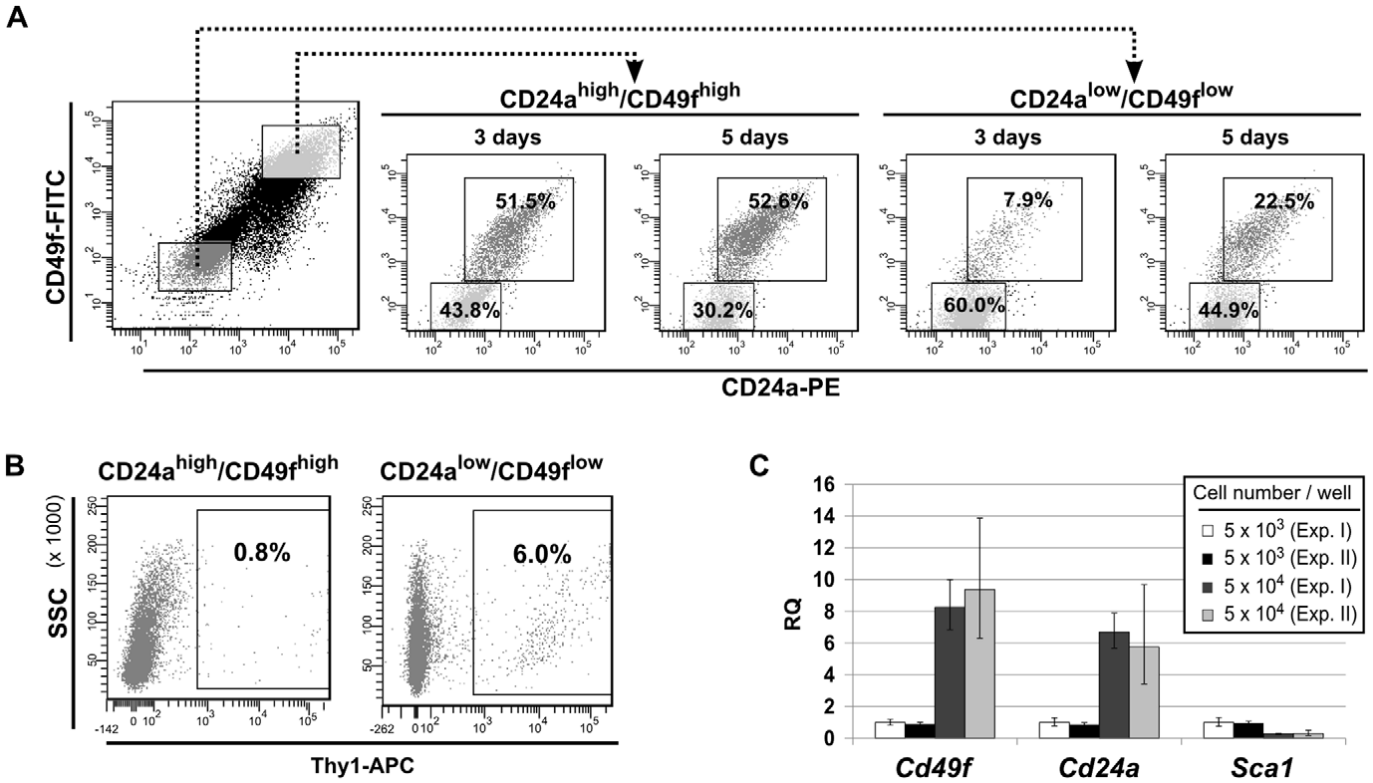
Repopulation activity of G-2 cell subsets differing in expression of CD24a/CD49f. (**A**) Repopulation activity of G-2 cell subsets differing in expression of CD24a and CD49f. CD24a^high^/CD49f^high^ and CD24a^low^/CD49f^low^ subsets were gated to exclude any overlap (left FACS dot plot). 5×10^4^ sorted cells were transferred back into culture (6-well plates) and the composition of the cell culture was re-analyzed for the same markers 3 and 5 days later by FACS. Note that in the resulting four FACS dot plots the gates of CD24a^low^/CD49f^low^ cells are restricted by the magnification. The percentages of events in each gate (quadrant) are given. (**B**) 5×10^4^ sorted CD24a^high^/CD49f^high^ and CD24a^low^/CD49f^low^ G-2 cells were transferred back into culture (6-well plates) and analyzed 5 days later for expression of Thy1 by FACS. The representative FACS dot plots are shown. (**C**) The CD24a^low^/CD49f^low^ subset was sorted and plated at low (5×10^3^ cells per well) and high (5×10^4^ cells per well) density in 6-well plates. After 5 days in culture, expression of *Cd49f*, *Cd24a*, and *Sca1* was quantitated by real-time qPCR. The experiment was performed in duplicate and repeated twice.

The data indicate that the Thy1^high^ cells represent a CSC-like subset in a G-2 culture, which in terms of hierarchy seems to be above the CD24a^high^ and CD24a^low^ subsets. However, the latter subsets also possess a repopulation activity: after 3 days in culture, the CD24a^high^/CD49f^high^ cells repopulated the CD24a^low^/CD49f^low^ subset to nearly the same ratio as initially measured in the parental G-2 culture (Figure 10A). Individually cultured CD24a^low^/CD49f^low^ cells repopulated the CD24a^high^/CD49f^high^ compartment with slower dynamics: 7.9% CD24a^high^ cells were detected after 3 days in culture, but their number increased by about 3-fold after additional 2 days (Figure 10A). Correspondingly, CD24a^high^/CD49f^high^ cells (Figure 10B, right) are less efficient than CD24a^low^/CD49f^low^ cells (Figure 10B, left) in regenerating the initial proportion of Thy1^high^ cells. Similar repopulation kinetics by individually cultured CD24a^high^ and CD24a^low^ subsets were observed using the G-2 derived cell clones (Figure S5).

The different kinetics for the accumulation of the complementary cellular subset in cultures of CD24a^high^/CD49f^high^ and CD24a^low^/CD49f^low^ cells, respectively, pointed to a potential role of intercellular communication. To substantiate this idea, 5×10^3^ and 5×10^4^ FACS-sorted CD24a^low^/CD49f^low^ G-2 cells were plated per well, and after 5 days in culture expression of the *Cd24a*, *Cd49f* and *Sca1* genes was quantified by qPCR. While the RNA levels of the *Cd24a* and *Cd49f* genes positively correlated with increased cell density, expression of the *Sca1* decreased as a function of cell density (Figure 10C).

**Figure 11:**
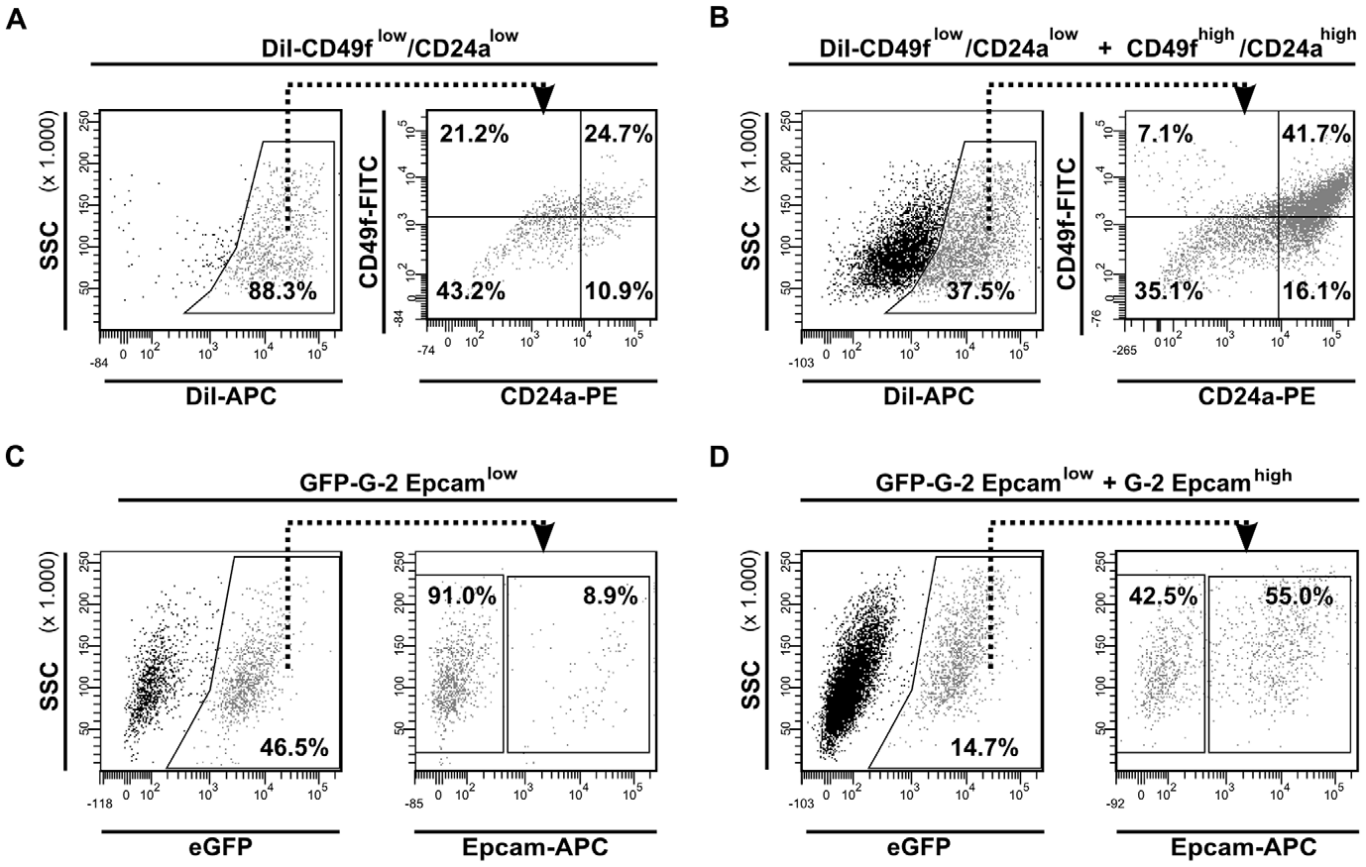
Repopulation activity of G-2 cell subsets depends on cellular interactions. (**A, B**) CD24a^low^/CD49f^low^ cells were labeled with DiI and 1×10^4^ labeled cells were either cultured separately (**A**), or plated at a 1∶1 ratio with non-labeled CD24a^high^/CD49f^high^ cells (**B**) in 6-well plates. After 3 days in culture, DiI labeled cells were analyzed for expression of CD24a and CD49f by FACS. (**C, D**) 1×10^4^ cells of the Epcam^low^ subset expressing eGFP were either cultured alone (**C**) or plated at a 1∶1 ratio with G-2 cells lacking eGFP from the Epcam^high^ subset (**D**) in 6-well plates. After 3 days in culture, GFP positive cells were analyzed for Epcam expression by FACS. The representative FACS dot plots are shown.

The importance of intercellular communication in subset repopulation was further supported by co-culture experiments. The CD24a^low^/CD49f^low^ subset was labeled with the lipophilic dye DiI and 1×10^4^ labeled cells were either cultured separately, or were plated at a 1∶1 ratio with non-labeled CD24a^high^/CD49f^high^ cells. After 3 days in culture, the DiI-positive cells (Figure 11A and B, left) were analyzed by FACS for expression of CD24a and CD49f (Figure 11A and B, right). Whereas 24.7% of the separately cultured DiI labeled CD24a^low^/CD49f^low^ cells became spontaneously positive for CD24a and CD49f (Figure 11A, right), this fraction increased to 41.7% during co-culture with non-labeled CD24a^high^ cells (Figure 11B, right). In a complementary experiment, the subset of eGFP-expressing Epcam^low^ G-2 cells was cultured either alone (1×10^4^ cells per well) (Figure 11C, left), or mixed in a 1∶1 ratio with non-transfected Epcam^high^ G-2 cells (Figure 11D, left). After 3 days in culture, 8.9% of the eGFP-positive cells revealed strong expression of Epcam (Figure 11C, right), while the fraction of Epcam-expressing eGFP-positive cells increased ∼4.5 times upon co-culture with non-labeled Epcam^high^ G-2 cells (Figure 11D, right).

#### c) Metabolic markers

**Figure 12:**
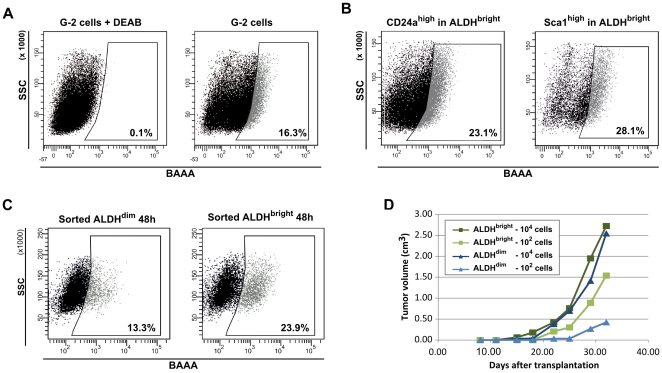
Properties of G-2 cell subsets differing in aldehyde dehydrogenase activity. (**A**) Representative FACS dot plots showing ALDH activity of G-2 cells (**A**, right panel) measured with the Aldefluor reagent (BAAA). To define the ALDH^bright^ gate, G-2 cells were stained with the Aldefluor reagent in presence of the ALDH inhibitor DEAB (**A**, left panel). (**B**) Representative FACS dot plots showing the expression of CD24a (**B**, left panel) and Sca1 (**B**, right panel) in the ALDH^bright^ subsets. Fluorescence-values of the Aldefluor-channel (FITC) and the CD24a-channel (PE) were compensated before analysis of the CD24a^high^ and Sca1^high^ fluorescence. (**C**) Repopulation activity of the ALDH^bright^ and ALDH^dim^ subsets. 5×10^5^ FACS-sorted ALDH^bright^ and ALDH^dim^ cells were individually plated in 6-well plates. After 2 days in the culture, cells were analyzed for ALDH activity by Aldefluor staining and FACS. (**D**) The graph shows growth curves of tumors arising upon transplantation of ALDH^bright^ and ALDH^dim^ G-2 cells into the left abdominal mammary gland of virgin syngeneic mice (WAP-T-NP8). The dark and light curves, respectively, represent tumor growth kinetics of 10^4^ and 10^2^ transplanted cells in individual recipient mice.

High ALDH activity has been described as a marker for normal stem/early progenitor cells in different organs in human and mice, and proposed to be a specific marker for normal and malignant human mammary stem cells [42]. We therefore tested G-2 cells for ALDH activity using the Aldefluor® reagent. The efficient retention of the Aldefluor® reagent (Bodipy-aminoacetaldehyde; BAAA) after conversion into the charged product BAA (BODIPY-aminoacetate) distinguished around 12–17% of the G-2 cells (ALDH^bright^, high BAA fluorescence) from intermediate and low (ALDH^dim^) fluorescing G-2 cells (Figure 12A, right panel). The specificity of this reaction was verified by applying the ALDH inhibitor DEAB, which completely blocked BAAA conversion (Figure 12A, left panel). Combining the Aldefluor® reaction with the staining of cell surface markers, a significant overlap between the CD24a^high^ (Figure 12B, left panel) and Sca1^high^ (Figure 12B, right panel) subsets and ALDH^bright^ and ALDH^dim^ cells was detected, indicating that the ALDH^bright^ and ALDH^dim^ compartments represent a mix of CD24a^high^ and CD24a^low^ subsets. To test the repopulation properties of ALDH^bright^ and ALDH^dim^ populations, FACS-sorted cells were propagated separately (5×10^5^ cells per well) and then re-analyzed by FACS using the Aldefluor® reagent. After 2 days in culture the ALDH^bright^ (Figure 12C, right panel) and ALDH^dim^ (Figure 12C, left panel) G-2 cellular subsets were able to almost perfectly regenerate the initial profile of ALDH activity in the G-2 culture, although the repopulation activity of ALDH^bright^ cells seemed to be slightly slower. In line with the *in vitro* data, transplantation of ALDH^bright^ and ALDH^dim^ subsets did not reveal substantial differences in their tumorigenic activity (Figure 12D). Furthermore, no significant differences in the tumorigenic activity was observed between ALDH^bright^ and ALDH^dim^ primary WAP-T tumor cells, and tumors arising from transplanted cells were similar to their parental tumors in histology and expression of epithelial markers (data not shown). The findings thus suggest that ALDH activity is not a suitable cancer stem cell marker in WAP-T mammary carcinomas.

#### d) Colony forming activity

As colony formation is considered to be a criterion inherent to stem cells, G-2 cells and cells of the G-2 cell derived clones C9 and C11, respectively, were seeded in soft agar (10 and 100 cells per well). Table 2 shows that all tested cells have a high capacity for clonal growth, as about 80% of the cells formed colonies. About 50–70% of the colonies could be expanded in culture, while the rest did not grow.

**Table 2:** Colony forming potential of G-2 cells and clones*.

	Colony forming cells (%)	
Cell line	10 cells	100 cells
G-2	86.7±11.5	87.3±6.8
G-2C9	83.8±5.8	91.0±4.6
G-2C11	80.0±10.0	87.0±5.3

*10 and 100 cells were seeded in soft agar (6-well plates) in triplicates. The colonies were counted after 2 weeks.

### The repopulation activity of G-2 cells is regulated by differentially expressed sets of transcription factors

**Figure 13:**
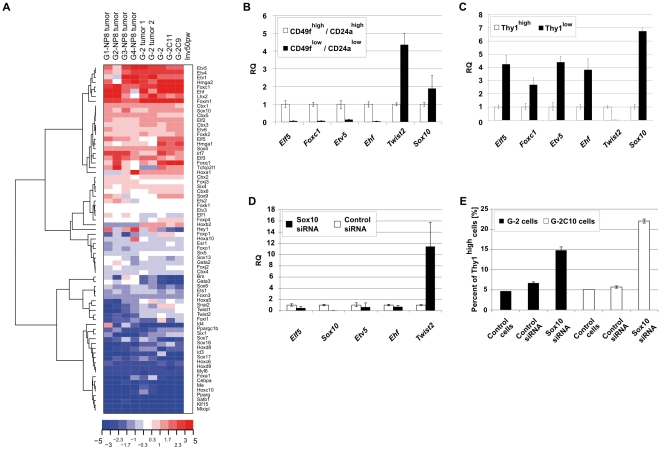
Expression of selected transcription factors in G-2 cells. (**A**) Hierarchical cluster analysis of microarray gene expression data. 73 genes coding for transcription factors (Table S1) were selected to generate a heat map. Gene expression log2-ratios are displayed with gene expression intensities of the mammary gland of a parous BALB/c mouse (50 days post weaning; Inv50pw) serving as reference. The relative expression values are color coded: red – high expression, blue – low. (**B–D**) Relative quantitation of gene expression by real-time qPCR in CD24a^high^/CD49f^high^ and CD24a^low^/CD49f^low^ (**B**), Thy1^high^ and Thy1^low^ (**C**) G-2 cell subsets, and G-2 cells transfected with Sox10 or control siRNA (**D**), respectively. Each assay was done in triplicate, and raw RQ values were calculated by normalizing to the *Gapdh* gene. (**E**) siRNA-mediated Sox10 depletion increases the number of Thy1-expressing cells. G-2 and G-2C10 cells were transfected with Sox10 or control siRNA and were analyzed for Thy1 expression by FACS after 5 days culture. A representative analysis is shown.

We hypothesized that the repopulation activity of G-2 cell subsets depends on the transcriptional competence of each subset, derived primarily from the expression of certain transcription factors (TFs). To substantiate this hypothesis, we first compiled a list of TFs (total of 73; see Table S1) selected by literature screening and by their expression in G-2 cells, in normal tissue, and in tumor samples. Hierarchical clustering analysis (Figure 13A) demonstrated the high similarity in TF expression between G-2 cell cultures and tumor samples and revealed two large gene clusters containing genes whose expression in comparison to normal mammary gland is either significantly reduced, e.g. *Esr1*, *Cebpa*, *Sox17*, *Sox18*, *Hoxc6*, *Id4*, *Pparg*, and *Foxa1*, or enhanced, e.g. *Ehf*, *Elf5*, *Etv1*, *Etv4*, *Etv5*, *Hmga1*, *Foxc1*, *Foxm1*, *Sox10* and *Tcfcp2l1*. We considered that the TFs from the latter group are essential for differentiation into the phenotypic subsets observed in G-2 cultures. As shown by qPCR analysis, expression of known regulators of normal and tumor mammary epithelial cell fate and function, namely *Ehf* [43], *Elf5* [44], *Etv5* [45], and *Foxc1* [46] is stronger in the CD24a^high^ subset (Figure 13B), whereas the CD24a^low^ subset is distinguished by a higher expression of the *Twist2* gene (Figure 13B), whose product has a function in EMT [38]. Notably, the expression of *Sox10*, a member of the SOX (SRY-related HMG-box) family of TFs, was significantly increased in the Thy1^low^ compared to the Thy1^high^ subset (Figure 13C), but was only slightly higher in the CD24a^low^ subset as compared to the CD24a^high^ subset (Figure 13B). The finding suggested a role for Sox10 in regulating the differentiation state of G-2 cells. Indeed, siRNA mediated depletion of *Sox10* resulted in a significant up-regulation of *Twist2*, but in no or only a slight regulation of epithelial-specific TFs (Figure 13D), indicating that Sox10 probably has a function in dampening EMT progression in G-2 cells via repression of *Twist2*. In support, 5 days after transfection of cells with Sox10 siRNA, the proportion of Thy1^high^ cells increased several folds as measured by FACS (Figure 13E). In conclusion, we identified Sox10 as a transcription factor controlling the differentiation states of G-2 cells.

## Discussion

**Figure 14:**
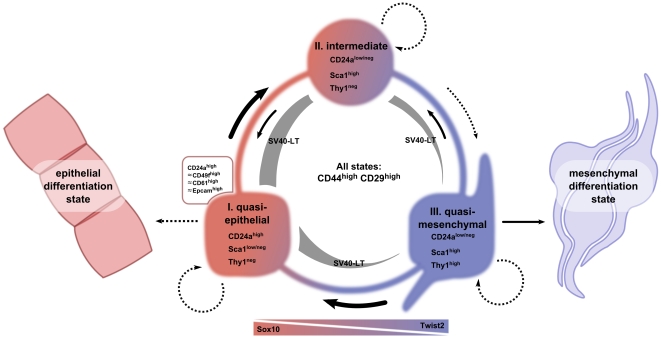
Schematic overview of the G-2 “cancer cell system”. The cellular composition of the assumed G-2 cancer cell system is determined by three interconvertible differentiation states: quasi-epithelial, intermediate, and quasi-mesenchymal. These states are characterized by the expression of a combination of cell surface associated proteins (CD24a, CD49f, CD61, Epcam, Sca1, Thy1), a specific set of transcription factors and by their cytoskeletal composition (Krt14, Krt18, vimentin), as well as by their ability to self-renew. Cells in all three states express the CD44 and CD29 proteins on their surfaces. The existence of the (self-sustaining) G-2 system depends on the expression SV40 LT, driven by the WAP-promoter, which is under control of epithelial transcription factors (TFs). Shut off of epithelial TFs leads to loss of SV40 LT expression and irreversible transition to a completely mesenchymal differentiation state. The existence of an irreversible epithelial differentiation state has not been proven, however, cannot be excluded. Transition rates between differentiation states and self-reproduction are determined by kinetic parameters which depend on intercellular communications and/or autocrine/paracrine factors as well as on the activity of the inversely expressed transcription factors (e.g. Sox10 and Twist2). The variable width of arrows should illustrate the observed differences in rates of transitions, e.g. the transition into quasi-mesenchymal is a rare (unfavorable) event, whereas the reverse transition readily takes place in culture. The figure was drawn using an open-source vector graphics program Inkscape.

The current CSC model provides a conceptual framework for studying tumors as cellular systems that in many aspects resemble normal tissues. In this regard, the evolution of the concept for normal stem cells also has implications for the CSC model. In normal tissues, the hierarchical organization and irreversible commitment for distinct lineages has been disputed. As an alternative, it has been suggested that phenotypic plasticity is a basic property of the stem cell state [47]. Extending the meaning of plasticity, it has been questioned that normal and cancer stem cells exist as an entity defined by discrete molecular properties, but rather together with the population of committed progenitors and their differentiated progeny comprise a homeostatic “stem cell system” where the cellular composition is regulated by feed-back mechanisms [10]. Our and other studies [48]–[50] suggest that, at least in established clonal cell cultures, but most likely also in tumor tissue, heterogeneity of differentiation states is an intrinsic property of what we term the “cancer cell system” (CCS) (Figure 14). We propose that the G-2 CCS is mainly populated by cells in three differentiation states: quasi-epithelial, intermediate, and quasi-mesenchymal. The differentiation states are operationally distinguished by a combination of cell surface associated proteins, a specific set of transcription factors and by the composition of cytoskeletal intermediate filaments, and are associated with the ability for self-renewal and uni- or bi-directional interconversion. We assume that transitions between differentiation states and self-renewal within the G-2 CCS are regulated by intercellular communications, autocrine/paracrine signaling and metabolic parameters (e.g. oxygen and metabolites supply). We expect that combinations and individual contribution of regulatory factors and circuits are different under *in vitro* and *in vivo* settings; however, their interplay results in the formation of comparable CCSs.

### “Cell of tumor origin” defines the cancer cell system

We think that in any CCS, the variability of differentiation states is determined by the origin of the tumor cells from certain tissue-restricted “founder” cells. As transforming events may hit any cellular compartment in the mammary epithelial cell hierarchy [51], but most likely the stem cell compartment [52], one has to expect that different scenarios of tumor initiation and progression can be realized and, consequently, different CCS can be created. For example, expansion of transiently existing or minor populations of gradually committed progenitors will result in the generation of differently populated CCS, which due to their origin from the same “stem cell system” may be equipped with many related features, like expression of differentiation-specific genes, but nevertheless deviate in their biological behavior. An illustration for a tumorigenic expansion of a minor population is the finding that in “luminal”-type *Wap-Cre;EN* mammary carcinomas [24] and low-grade WAP-T tumors (this study), the embryonic K8^+^K14^+^ cellular subset [53] is significantly expanded, while in the adult normal mammary gland K8^+^K14^+^ cells constitute a rare population that overlaps with CD61^+^ luminal progenitors [24]. Expansion of a cell population expressing a mix of “basal” and “luminal” cytokeratins was also observed in mammary epithelium with impaired Notch signaling [54] and depleted of the PDZ domains-containing scaffold protein Par3, which regulates cell polarity [55].

The co-expression of vimentin and cytokeratins in individual cells in high-grade WAP-T tumors and in G-2 cultures probably can also be attributed to the expansion of transformed, vimentin-expressing cells normally present in the mammary epithelial “stem cell system”. Indeed, significant vimentin expression was detected in the MRU (mammary repopulating unit) cell population that is responsible for mammary tissue regeneration upon transplantation into the cleared fat pad [56]. The MRU subset is a rare population of mammary epithelial cells co-expressing CD24a and CD49f, but lacking Sca1expression [56]. Since vimentin is expressed during mammary morphogenesis [57] and is associated with a motile phenotype [58], it is conceivable that vimentin marks a subset of non-constrained cells required for tissue maintenance. The predominant intermediate differentiation state of high-grade WAP-T tumors likely results from the oncogenic transformation of this cellular subset. In such a CCS, the ability for bidirectional interconversion (plasticity) between differentiation states may account for a better adaptability to microenvironmental conditions and eventually provide a basis for tumor aggressiveness (e.g. rapid growth and metastatic behavior).

The use of tissue-specific promoters in transgenic mouse models of mammary tumors, e.g. WAP and MMTV, limits the cellular compartments of the mammary “stem cell system” that can be deregulated and transformed by oncogene expression. This results in the outgrowth of phenotypically similar tumor types, which, however, may differ in their genetic and epigenetic traits and more importantly, represent different CCSs. Therefore, it is not surprising that tumorigenic subpopulations in mammary carcinomas induced by different oncogenes, driven by different promoters or induced by loss of tumor suppressors in mammary epithelial cells are differing in their profiles of cell surface markers: e.g. the Sca1^+^ subset in BALB-neuT mice [37], the CD29^H^CD24^H^ subset in p53-null mammary tumors [30] and in mammary tumors from BRCA1 conditional knockout mice [35], and the CD61^+^ subset in MMTV-*Wnt-1* mice [36]. A related ambiguity of cell surface markers (e.g. Sca1) for the enrichment of CSCs has been recently reported for three mouse models of lung adenocarcinoma [59]. In line with these observations, it is also questionable to generally associate other stemness-related properties, e.g. drug efflux activity that characterizes a so-called “side population”[60] and high aldehyde dehydrogenase activity [61] with the tumorigenic potential. In G-2 cell cultures and primary cells purified from WAP-T tumors, for example, no “side population” as measured by efflux of the Hoechst dye could been observed (data not shown), and no correlation was found between metabolic Aldefluor conversion and tumorigenic activity. Under these circumstances the application of cell surface or metabolic markers maybe operationally useful for the enrichment of distinct cellular subsets. However, without knowledge about the function of these markers in the respective cellular context it is difficult to conceive the biological properties of these cellular subsets, and to assess their association with a CSC potential and their position in the respective CCSs.

### Differentiation states within “cancer cell system” in cell culture and *in vivo*

According to our definition, the term “cancer cell system” is applicable to cells growing in culture as well as to cells forming primary and transplanted tumor. Due to the inherent differences between *in vitro* and *in vivo* conditions some limitations must be considered that may cause significant variations in the composition and properties of a CCS. In cell culture, irreversibly differentiated cells inevitably get lost during cell passage, as terminal differentiation presupposes an exit from the cell cycle, whereas *in vivo* terminally differentiated cells remain in the tumor and may contribute to the formation of the stromal compartment. Thus cell culture leads to the selection of actively proliferating cells capable of generating viable progeny under cell culture conditions. This may explain the gene expression differences measured between G-2 cell culture and tumor samples. In this respect, it is likely that the pronounced co-expression of Krt14 and Krt8/18 proteins in G-2 culture, but not in G-2 tumors, is attributed to selection in cell culture for a pro-proliferative function of Krt14. Such a proliferation-promoting role of Krt14 has been demonstrated in transgenic mice overexpressing Krt14 in pulmonary epithelium [62]. However, how cytoskeletal Krt14 regulates cellular proliferation is not yet known. Interestingly, during primary cell culture of normal mammary epithelial cells a transition to co-expression of both lineage-specific cytokeratins occurs in colonies derived from CD24^high^ cells, which before plating expressed only the “luminal” cytokeratins [63].

In culture, the G-2 CCS is populated by proliferating cells in quasi-epithelial, intermediate, and quasi-mesenchymal differentiation states (Figure 14). The latter state is represented by expression of Thy1, is reversible and can most likely transit directly into a quasi-epithelial state. How the Thy1-positive cells are (re-)generated in G-2 culture depleted by FACS of Thy1-expressing cells is unclear and needs to be explored. We speculate that under certain circumstances (e.g. at low plating density) the transition from an intermediate to a quasi-mesenchymal state may take place (Figure 14). Thy1, a marker of myoepithelial and fibroblastic cells [64], [65], has been linked to mammary CSCs, as a small subset of Thy1^+^/CD24^+^ cells, comprising 1%–4% of the tumor cells purified from mammary carcinomas in MMTV-*Wnt*-*1* mice, has been found to behave like CSCs [33]. In the G-2 cell culture, Thy1 expression characterizes a subset of cells mostly located within the CD24a^low^/Sca1^high^ compartment. However, roughly 5% of Thy1^high^ cells overlap with the CD24a^high^ subset. These double-positive (Thy1^high^/CD24a^high^) cells might be related to the corresponding subset in MMTV-*Wnt*-1 tumors; however, their paucity in G-2 cultures argues against this. Noteworthy, in WAP-T tumors Thy1-expressing cells are mostly located within or close to fibroblastic stroma, which likely originates from recruited normal mesenchymal stem cells [66] and probably provides a seeding niche for tumor cells undergoing mesenchymal differentiation. Future studies of Thy1-expressing cells in G-2 culture and WAP-T tumors should provide new insights into their role in the phenotypic composition of CCSs.

Emerging evidence indicates that the host microenvironment, represented by a combination of immunologic, trophic and local humoral factors, not only governs tumor growth but also the differentiation states of tumor cells. For instance, in transplanted, non-transgenic mice CD8^+^-T lymphocytes stimulated an incomplete EMT of transplanted mammary epithelial tumor cells [67]. It is assumed that immunoediting resulted in the selection of tumor cells that lack expression of the transgene (MMTV-promoter driven neu protein) and display an intermediate differentiation state, which however can be reversed back to an epithelial state upon re-transplantation into syngeneic mice [68]. In our model, we observed that G-2 tumors grown in non-transgenic BALB/c mice display a mesenchymal phenotype; however, tumor cells retained expression of the WAP-promoter driven SV40 LT protein. The identity and properties of these cells, especially the contribution of immune cells to the phenotypic plasticity of transplanted G-2 cells, is a matter of ongoing studies.

Furthermore, tumor vascularization not only serves for transport of metabolites and humoral factors, but also provides another regulatory mechanism of tumor cell differentiation based on the emerging role of oxygen. Depending on local oxygen concentration the differentiation state of tumor cells is adjusted to one that is more adequate with respect to metabolic requirements. Upon moderate hypoxic conditions epithelial tumor cells undergo EMT [69], which is transcriptionally regulated by Hif-1alpha and its downstream target Twist1 [70]; conversely, hyperbaric oxygen treatment triggers a reversed process, MET [71].

As immune cells are absent in culture and oxygen supply is constant, it is obvious that the differentiation states of G-2 cells are regulated by intrinsic factors. In G-2 culture around 80% of the cells are colony-forming, but not each colony will form a CCS (around 50% of G-2 cell colonies are CCS-forming). Assuming that a newly formed colony is composed of nearly identical cells which with respect to their differentiation state recapitulate the phenotype of the mother cell, possibly a restriction point controls the transition from a monomorphic colony to a heterogeneous CCS. The number of cells in a colony might be crucial for the transition into a more complex state where cells in different differentiation states cooperate to maintain the whole system. When a certain critical concentration of cells and secreted factors is reached, the transit of a colony from one state to another may be triggered. In support of this idea, we demonstrate that co-culture of complementary cellular subsets greatly influences the repopulation activity of their counterparts. Although we did not address in this study the question which signaling pathways are involved in the regulation of the G-2 CCS, we observed in preliminary experiments that the differentiation states of G-2 cells are influenced by multiple, synergistically or antagonistically acting pathways including interferon-, BMP- and HGF-mediated signaling cascades. Further work is needed to explore the contribution of these pathways in more details.

Consequently, we propose that *in vivo*, upon transplantation of single cells, the probability for tumor outgrowth is determined by the generation of a minimal CCS, which is determined by a combination of intrinsic (e.g. intercellular communications) and external factors (e.g. interactions with the immune system and supply of oxygen). Similarly, we think that establishment of a stable “cancer cell system” at the seeding site most likely is the decisive event for the outgrowth of a metastatic tumor, where similar as in the development of the primary tumor, mesenchymal and epithelial cells cooperate to create a niche and generate a proliferative cell pool.

### Role of the gene regulation network in the maintenance of a “cancer cell system”

In terms of mathematical formalism, any cellular system is maintained via a metastable state of a gene regulation network (GRN) that at the level of a single cell is proficient to restore the phenotypic heterogeneity of the whole system [72]. The key components of any GRN are transcription factors, with their combination shaping the phenotype of a single cell and the behavior of any cellular system. TFs regulate the differentiation state and biological properties of tumor cells and represent possible targets for cancer therapy. The activity of a single TF may profoundly change the tumor cell phenotype, as for example, RNAi-mediated knockdown of Klf17 (Krüppel-like factor) promotes EMT and lung metastasis of 168FARN cell line, whereas overexpression of Klf17 in the metastatic 4T1 cell line considerably limits their metastatic potential [73]. In a related study Twist1 was identified as a positive regulator of the metastatic behavior of 4T1 cells [74]. It is noteworthy that 4T1 and 168FARN are cell lines derived by different culture protocols from a single mammary tumor spontaneously arising in BALB/cfC3H mouse [75]. These cell lines provide an example of how the differences in GRN composition contributes to creation of a distinct cellular phenotype in a context of related genetic background.

In the present work we showed by qPCR that FACS-separated G-2 cellular subsets differ in the transcription of genes linked to the respective subset, e.g. *Cd24a*, *Cd49f*, *Sca1*, and do express, though at different levels, the transcription factors associated with epithelial (e.g., Ehf, Etv5 and Elf5) and mesenchymal (e.g., Twist2) differentiation gene expression programs. Expression of these TFs obviously provides the basis for a competent transcriptional network, which specifies and maintains the self-reproducing G-2 CCS. As a notable component of the G-2 GRN we identified the transcription factor Sox10, known as a glial and neural crest cell fate regulator [76], and demonstrated that perturbation in the expression of a single component of the GRN can significantly influence the composition of the CCS. The surprising function of Sox10 in the regulation of EMT/MET in G-2 CCS as well as the transcriptional targets of Sox10 remains to be elucidated. Also the question whether *Twist2* is transcriptionally regulated directly or indirectly by Sox10 is still open and needs further investigations.

An important prerequisite for the interconversion between complementary subsets is a transcriptional permissiveness of those genes whose transcription characterizes the respective differentiation state. The creation of the permissive state of a differentiation-specific gene might rely on mechanisms related e.g. to activation of primary response genes, which are regulated at the level of transcript elongation and processing [77]. Alternatively, transcription of differentiation-specific genes might be attenuated, but still weakly active and rapidly switched to a more active state upon activation of respective signaling cascades, initiating and accompanying the transition into another differentiation state. In this scenario, the regulation of transcriptional activity might be exerted by transcription factors acting in concert with signaling pathways. For instance, a transient inflammatory signaling cascade activated by Src kinase in human MCF10A cells triggers a Lin28B/let-7 mediated epigenetic switch resulting in engagement of transcription factor STAT3 and acquirement of fully transformed phenotype and CSC properties [78].

In a recent study related to our work, an identical epigenetic state in the promoter region of the *CD24* gene has been observed between interconvertible CD24-positive and negative subsets of CD44^+^ breast cancer cells [50], indicating that transcriptional permissiveness is a general phenomenon. It remains to be elucidated how the specific set of transcription factors in G-2 cells regulates the differentiation states and how the signaling cascades are crossed with the activity of transcription factors.

### Conclusion

The main challenge in tumor therapy remains to eradicate all cancer cells. Thus the efficiency of a treatment strategy not only depends on the understanding of the genetics and epigenetics of single cancer cell or of distinct cellular subsets which might behave like CSCs, but also on the knowledge of the mechanisms which determine the behavior of the entire cancer cell population as a dynamic, self-reproducing system.

## Materials and Methods

### Ethics statement

All mice were housed under SPF conditions in accordance with official regulations for care and use of laboratory animals (UKCCCR Guidelines for the Welfare of Animals in Experimental Neoplasia) and approved by Hamburg's Authority for Health (Nr. 88/06).

### Establishment and propagation of G-2 cell culture

Singularized primary tumor cells were transferred into culture according to a procedure published for normal mammary epithelial cells [79]. All attempts to isolate a stable, SV40-LT expressing epithelial cell line from WAP-T mice derived tumors were unsuccessful, as during first days in culture the cells progressively lost WAP-promoter dependent SV40-LT expression and acquired a fibroblastic phenotype, as also described by others [80]. However, we succeeded in establishing an epithelial cell culture (termed G-2 cells) from a tumor of a WAP-TxWAP-mutp53 bi-transgenic mouse [16], [18]. Primary cells were propagated in DMEM/10% FCS medium supplemented with 5 µg/ml insulin, 5 µg/ml hydrocortisone, 5 µg/ml prolactin, and 5 µg/ml β-estradiol (all from Sigma) at 37°C, 5% CO_2_. Starting from the 10th passage the cells were cultured in DMEM/10% FCS medium without hormones and split twice per week at a 1∶3 ratio.

### Agar cloning

Seeding and culture of cells in soft agar was performed according to standard procedures. After 2–3 weeks in the incubator, colonies were counted, and single colonies were transferred into 24-well cell culture dishes using sterile pipette tips. Ten colonies from two independent experiments, C5, C9, C10, C11, C13, K1, K2, K6, K7, and K8, were expanded into stable cell lines.

### siRNA transfection

Sox10 siRNA (XM_128139si.2; sense strand: 5′-AAGGACCAUCCGGACUACA-3′) and control siRNA (sense strand: 5′-CGAACUUUUGGACGCGCAC-3′) were obtained from Eurofins MWG Operon. siRNA transfections were performed in 6-well cell culture plates using Oligofectamine™ (Invitrogen) according to manufacturer's protocol.

### Orthotopic transplantation

#### a) Preparation of primary cells

Tumor pieces were minced with the help of 2 scalpels under sterile conditions, washed with Quantum 286 medium (PAA) and treated with enzymes (200 U/ml collagenase III (Worthington) and 100 U/ml hyaluronidase type I-S (Sigma) in Quantum 286 medium) for 2–4 h at 37°C under shaking. After washing with phosphate-buffered saline (PBS) buffer, the cells were first treated for 5 min with 0.25% Trypsin-EDTA (Biochrom) at 37°C, followed by 15 min treatment with dispase/DNaseI solution (20 U/ml dispase (Worthington) and 100 U/ml DNase I (Sigma) in Quantum 286 medium) at 37°C. Cells were then resuspended in 5 ml MACS-buffer (0.5 % BSA, 2 mM EDTA in PBS, pH 7.2) and sequentially filtrated through a coffee mesh, 80 µm and 30 µm nylon filters (Reichelt). After counting, the cell concentration was adjusted for lineage depletion according to the manufacturer's protocols (lineage depletion kit, Miltenyi). Cell viability was assessed by Trypan blue staining (Invitrogen).

#### b) Transplantation

Primary tumor cells or G-2 cells, respectively, were resuspended in 20 µl of a 1∶1 mixture of Quantum 286 medium and BD Matrigel Matrix High Concentration (HC), Growth Factor Reduced (GFR) (BD Bioscience; Cat. No. 354263) and kept on ice until transplantation. 8 to 16 weeks old virgin WAP-T-NP8 mice were anesthetized by intra-peritoneal injection of 7–8 µl ketamine/xylazine per gram (12 mg/ml ketamine, 1.6 mg xylazine in 0.9% NaCl solution) and 1.2 µl carprofen per gram (50 mg/ml carprofen, Pfizer) was subcutanousely injected as analgesic. After a 1–2 mm incision of the skin, the cell suspensions were injected with a 0.3 ml Micro-Fine syringe (BD Bioscience, Cat. No. 4144150) into the left abdominal mammary gland and the wound was sutured. The operation was performed under sterile conditions. Size of growing tumors was measured twice a week with a caliper.

### Histology

Tissue specimens were fixed at room temperature overnight with 4% formaldehyde in 0.1 M phosphate buffer solution (pH 7.3), washed for 4–6 hours in 0.1 M phosphate buffer and stored thereafter in a 50% EtOH solution at 4°C. Fixed tissue specimens were embedded in Paraplast X-TRA (Sherwood Medical) and deparaffinized sections were stained with hematoxilin and eosin. Digital pictures were taken with a Zeiss Axioskop 2 combined to a CCD microscope camera “ProgRes C12” (Jenoptik).

### Immunofluorescence staining

#### a) Tumor cryosections

Pieces of tumor tissues were embedded in Shandon cryomatrix™ (Thermo Scientific) immediately after dissection, frozen on a mix of dry ice/isopentan and conserved at −80°C. 7–8 µm cryosections were made at −20°C in a Leica CM3050 cryostat, collected on SuperFrost slides (Thermo Scientific) and immunostained (the list of primary antibodies is in Table S2). Secondary antibodies were purchased as Alexa® Dye or DyLight® conjugates from Invitrogen and Dianova.

#### b) Adherently growing cells

1×10^5^ cells were plated on glass coverslips in 6-well plates, fixed with 4% paraformaldehyde (PFA) in PBS, and immunostained (the list of primary antibodies is in Table S2). Secondary fluorochrome-coupled antibodies were obtained from Invitrogen.

Nuclei were stained either with DRAQ5 (Biostatus), DAPI (Sigma) or TO-PRO®-3 iodide (Invitrogen) and mounted with Mowiol 4–88 (Merck). Images were captured as Z-stacks using an Axiovert 200 microscope equipped with a LSM 510 META confocal scanner (Carl Zeiss MicroImaging GmbH). Raw data were exported to the Huygens Essential software (version 2.7.2p0, Scientific Volume Imaging B.V.) and deconvoluted. The restored image data sets were visualized and processed with the Imaris software package (version 4.1.3, Bitplane AG). Colocalization was calculated with the ImarisColoc module, and a map of the colocalized voxels was saved as separate channel.

### Flow cytometric analysis and fluorescence activated cell sorting

Flow cytometric analyses were performed on FACScanto (BD Bioscience) and fluorescence activated cell sorting (FACS) on a FACSaria (BD Bioscience). Gating parameters were established using negative controls. In the case of multiparameter analysis single stained samples were used to establish gating parameters.

#### a) Antibody-staining of cell surface markers

Primary or cultured cells were washed twice with MACS-buffer and resuspended at a concentration of 1×10^6^ cells per 100 µl in MACS-Buffer. Cells were labeled using the fluorescent dye conjugated antibodies (the list of antibodies is in Table S2). After 30–45 min incubation on ice, the cells were washed twice with MACS-buffer and resuspended at 1×10^6^ cells per 0.5 ml MACS-buffer and transferred into a 4 ml tube.

#### b) Intracellular staining

1×10^6^ cells were washed twice with PBS and fixed with 500 µl 80% EtOH for 10 min, washed twice with cold PBS/2 mM EDTA and permeabilized using 100 µl 1% Triton-X100 in PBS. After blocking with 500 µl 0.5% BSA/PBS for 30 min, cells were incubated with primary (anti-vimentin, Santa-Cruz; anti-keratin K18, Progen Biotechnik) and Alexa488-, Alexa633-conjugated secondary antibodies (Invitrogen) in 100 µl 0.5% BSA/PBS for 30 min, washed twice with 0.1% Tween-20 in PBS/2 mM EDTA and resuspended in PBS/2 mM EDTA for flow cytometry analysis. To define gates, Fluorescence Minus One (FMO) controls were used and background fluorescence was excluded using secondary antibodies alone.

#### c) Cytospin preparations

5×10^4^ FACS-sorted cells were diluted in 300 µl PBS. Slides and filters were placed into appropriate slots in the cytospin (Cytospin 3; Shandon), with the cardboard filters facing the center of the cytospin. Cells were spun down at 500 rpm for 5 minutes. Filters were removed and slides were dried for a few minutes. Immunofluorescent staining was performed as described above.

#### d) DiI staining

Cells of the FACS-sorted CD24a^low^/CD49f^low^ subset were incubated with 2 µM DiIC18(5)-DS (Invitrogen) in PBS for 5 min at 37°C while shaking, and then for an additional 15 min at 4°C. The DiIC18(5)-DS labeling efficiency was close to 99%. After labeling, cells were washed twice with PBS, transferred into warm DMEM/10% FCS in 6-well plates and either cultured alone or co-cultured at the 1∶1 ratio with non-labeled cells of the CD24a^high^ subset.

#### e) Aldefluor® assay

To measure aldehyde dehydrogenase activity, the Aldefluor® kit (Stemcell technologies) was used following the recommendations of the manufacturer. Briefly, the cells were resuspended at 1×10^6^ cells per ml Aldefluor® buffer and 5 µl of activated ALDH substrate per 1×10^6^ cells were added. As negative control, 1 ml of the sample was treated with 5 µl of 1.5 mM DEAB (diethylaminobenzaldehyde), a specific ALDH inhibitor. The cell suspensions were rocked at 37°C for 40 min, washed with Aldefluor® buffer and finally resuspended in 0.5 ml buffer.

### Gene expression analysis

Total RNA was isolated from cells using TRIzol® (Invitrogen) and digested with RNase-free DNase I (Qiagen) according to the manufacturer's instructions. The quality and integrity of the total RNA was evaluated with the 2100 Bioanalyzer (Agilent Technologies). Labeling, hybridization on the Affymetrix microarray chips (MOE430 2.0) and image data processing were completed by the Signature Diagnostics AG (Potsdam) according to the Affymetrix standard protocol. The raw signals were background corrected and normalized using RMA procedure and quantile normalization (Bioconductor package simpleaffy version 1.16.0) on the R statistical platform (version 2.6.2). The differentially expressed genes were identified using a Welch approximation based t-test (package stats, version 2.6.2) followed by a Benjamini-Hochberg correction procedure. Hierarchical clustering of genes and samples was done using heatmap.2 procedure (package gplots, version 2.6.0), correlation distance with centroid linkage and visualized using TreeView-ver.1.60 software. The microarray gene expression data discussed in this paper have been deposited in MIAMExpress (http://www.ebi.ac.uk/microarray) and are accessible through E-MEXP-2669 accession number.

### Quantitative Real Time PCR

RNA was purified using the RNeasy Mini or Plus Micro Kits (Qiagen), and reverse transcribed with the High Capacity RT kit (Applied Biosystems). PCR was performed using the Power SYBR Green PCR Mastermix (Applied Biosystems) in a standard program running in an ABI 7500 Fast thermal cycler (Applied Biosystems). PCR reactions for each sample were repeated in triplicates. The integrity of the amplified products was confirmed by melting-curve analysis. PCR primers (see Table S2 for primer sequences) were selected from a Primer Bank (http://pga.mgh.harvard.edu/primerbank/index.html) or designed using Primer Designer 4 (Scientific & Educational Software). PCR efficiency was measured for each primer pair using serial dilution of cDNA. *Gapdh* was used as endogenous control. Relative quantitation of transcript levels with respect to the calibrator was done based on 2^−ΔΔC^_T_ algorithm.

### Lentiviral transduction

The SFFV-promoter in the LeGo-G lentiviral, eGFP-coding vector (kindly provided by Dr. Carol Stocking) was replaced by a sequence upstream of the *Wap* gene. Briefly, a 1,454 bp fragment of an upstream sequence flanking the *Wap* transcription start site was amplified by nested PCR (see for primer sequences in Table S2) from mouse genomic DNA and inserted into the BamHI/NotI cloning sites of the LeGo-G vector. 293T packaging cells were grown to around 50% confluence on a 10-cm dish. For virus production, third-generation packaging plasmids pMDLg/pRRE (3 µg), pRSV-Rev (2 µg), VSV-G (2 µg) (kindly provided by Dr. Carol Stocking, HPI) were mixed for co-transfection with 4 µg LeGo-G plasmid DNA and PEI transfection reagent (Polysciences) in 1 ml Optimem (Invitrogen) medium and incubated for 10 min. The medium of 293T cells was replaced by 5 ml Optimem containing the transfection mixture and cells were incubated 12 h at 37°C. The medium was exchanged with 5 ml DMEM/10% FCS and transfected cells were incubated for further 2 days. Cell culture medium containing viral particles was harvested, cleared through a sterile filter (pore size 0.45 µm), and stored at −80°C. For lentiviral transduction, G-2 cells were grown to around 50% confluence on 6-well plates. 3 days after transduction, eGFP expression was analyzed by live-cell fluorescence imaging in a Leica DMI6000 B microscope. eGFP-expressing cells were enriched by cell sorting.

## Supporting Information

Table S1 Excel datasheets containing data of gene expression microarray analysis and list of genes used for generation of heat maps.
(6.84 MB XLS)

Table S2 List of primers and primary antibodies used in this study.
(0.03 MB XLS)

Figure S1 Expression of intermediate filament proteins in cells of G-2 subclones. (A, B) Confocal images of cells of G-2 clones C9 (A) and C11 (B) stained with antibodies against keratin 14 (green) and keratin 18 (red). Nuclei were visualized with TO-PRO-3. Confocal sections were deconvoluted using Huygens Essential and processed with Imaris software. (C, D) 5 clones of the first G-2 cloning (C5, C9, C10, C11, and C13: late passages [P>10]) and 5 of the second cloning (K1, K2, K6, K7, and K8: early passages [P<3]) were subjected to real-time qPCR analysis for Krt14 (C) and Krt18 (D) expression. Gapdh was used as housekeeping gene and the respective results were calibrated on parental G-2 cell expression values. (E, F). 7 and 6 secondary clones, respectively, derived from primary clones G-2C9 (E: sC9-1, sC9-2, sC9-3, sC9-5, sC9-6, sC9-7 and sC9-8) and G-2C11 (F: sC11-2, sC11-3, sC11-4, sC11-6, sC11-7, and sC11-8) were subjected to real-time qPCR analysis for SV40-LT, Krt14 and Krt18 expression. Gapdh was used as housekeeping gene and the respective results were calibrated on sC9-1 and sC11-2 expression values. (G) Confocal images of G-2 cells and subclones G-2C5, G-2C9, G-2C10 and G-2C13 stained for vimentin (green). Nuclei were visualized with TO-PRO-3. Confocal sections were deconvoluted using Huygens Essential and processed with Imaris software. Scale bar: A and B: 20 µm; G: 30 µm.
(2.15 MB TIF)

Figure S2 Characterization of transplanted G-2 tumor in BALB/c wild-type recipient mouse. (A–D) Immunostaining of a G-2 tumor in BALB/c recipient mouse for (A) Epcam (red), (B) keratin 8/18 (red) and vimentin (green), (C) SV40-LT (red) and vimentin (green), and (D) keratin 14 (red). Residual structures of the normal mammary gland were observed in A (positive for Epcam), B (positive for keratin 8/18) and C (positive for keratin 14). Nuclei were visualized with DAPI. Scale bars: 200 µm.
(4.05 MB TIF)

Figure S3 qPCR analysis of G-2 subsets. (A, B) FACS-sorted CD49f^high^/CD24a^high^ and CD49f^low^/CD24a^low^ (A) or Sca1^high^ and Sca1^low^ (B) G-2 subpopulations were subjected to real-time qPCR analysis for SV40-LT, Krt14 and Krt18 expression. Gapdh was used as housekeeping gene and the respective results were calibrated on the expression values of CD49f^low^/CD24a^low^ and Sca1^low^ subpopulations. (C) Real-time amplification plots of CD49f^high^/CD24a^high^ and CD49f^low^/CD24a^low^ samples for Gapdh, Cd24a, Cd49f, and Sca1 shown on a logarithmic scale Delta Rn.
(0.87 MB TIF)

Figure S4 Characterization of the Thy1^high^ cell population of G-2 cells and subclones. (A) Representative FACS dot plots showing the expression of Thy1 in 5 clones of the first G-2 cloning (C5, C9, C10, C11, and C13: late passages [P>10]) and 2 of the second cloning (K1 and K2: early passages [P<3]). The gating was adjusted with the help of an antibody control. (B) Co-immunostaining of G-2 cells grown on coverslips for Sca1 (red) and Thy1 (green). Nuclei were stained with DAPI. (C) Thy1^low^ and Thy1^high^ G-2 subsets were FACS sorted and transcription levels of Cd24a and Cd49f genes were analyzed via real-time qPCR. Gapdh was used as housekeeping gene and results were calibrated on the expression values of the Thy1^low^ subpopulation. (D) Cytospin preparations of FACS-sorted Thy1^high^ and Thy1^low^ G-2C9 cells were stained for keratin 18 (D, upper panels) and vimentin (D, lower panels). Nuclei were stained with DAPI. Scale bar: C: 40 µm; D: 75 µm.
(2.37 MB TIF)

Figure S5 Repopulation activity of G-2K1 and G-2K2 cell subsets. Representative FACS dot plots showing the repopulation activity of two G-2 clones, G-2K1 (upper row) and G-2K2 (lower row), differing in the expression of CD24a and CD49f. CD24a^high^/CD49f^high^ and CD24a^low^/CD49f^low^ subsets were gated during cell sorting to exclude any overlap. 5×10^4^ sorted cells were transferred back into culture and the composition of the culture was analyzed 3 and 5 days later by FACS.
(1.04 MB TIF)

## References

[1] Rubin H (2007). Ordered heterogeneity and its decline in cancer and aging.. Adv Cancer Res.

[2] Dick JE (2008). Stem cell concepts renew cancer research.. Blood.

[3] Ward RJ, Dirks PB (2007). Cancer Stem Cells: At the Headwaters of Tumor Development.. Annu Rev Pathol.

[4] Lobo NA, Shimono Y, Qian D, Clarke MF (2007). The biology of cancer stem cells.. Annu Rev Cell Dev Biol.

[5] Nowell PC (1986). Mechanisms of tumor progression.. Cancer Res.

[6] Lotem J, Sachs L (2006). Epigenetics and the plasticity of differentiation in normal and cancer stem cells.. Oncogene.

[7] Quintana E, Shackleton M, Sabel MS, Fullen DR, Johnson TM (2008). Efficient tumour formation by single human melanoma cells.. Nature.

[8] Shackleton M, Quintana E, Fearon ER, Morrison SJ (2009). Heterogeneity in cancer: cancer stem cells versus clonal evolution.. Cell.

[9] Gupta PB, Chaffer CL, Weinberg RA (2009). Cancer stem cells: mirage or reality?. Nat Med.

[10] Lander AD (2009). The ‘stem cell’ concept: is it holding us back?. J Biol.

[11] Klymkowsky MW, Savagner P (2009). Epithelial-mesenchymal transition: a cancer researcher's conceptual friend and foe.. Am J Pathol.

[12] Polyak K, Weinberg RA (2009). Transitions between epithelial and mesenchymal states: acquisition of malignant and stem cell traits.. Nat Rev Cancer.

[13] Thiery JP, Acloque H, Huang RY, Nieto MA (2009). Epithelial-mesenchymal transitions in development and disease.. Cell.

[14] Mani SA, Guo W, Liao MJ, Eaton EN, Ayyanan A (2008). The epithelial-mesenchymal transition generates cells with properties of stem cells.. Cell.

[15] Morel AP, Lievre M, Thomas C, Hinkal G, Ansieau S (2008). Generation of breast cancer stem cells through epithelial-mesenchymal transition.. PLoS One.

[16] Heinlein C, Krepulat F, Lohler J, Speidel D, Deppert W (2008). Mutant p53(R270H) gain of function phenotype in a mouse model for oncogene-induced mammary carcinogenesis.. Int J Cancer.

[17] Jannasch K, Dullin C, Heinlein C, Krepulat F, Wegwitz F (2009). Detection of different tumor growth kinetics in single transgenic mice with oncogene-induced mammary carcinomas by flat-panel volume computed tomography.. Int J Cancer.

[18] Krepulat F, Lohler J, Heinlein C, Hermannstadter A, Tolstonog GV (2005). Epigenetic mechanisms affect mutant p53 transgene expression in WAP-mutp53 transgenic mice.. Oncogene.

[19] Schulze-Garg C, Lohler J, Gocht A, Deppert W (2000). A transgenic mouse model for the ductal carcinoma in situ (DCIS) of the mammary gland.. Oncogene.

[20] Tzeng YJ, Guhl E, Graessmann M, Graessmann A (1993). Breast cancer formation in transgenic animals induced by the whey acidic protein SV40 T antigen (WAP-SV-T) hybrid gene.. Oncogene.

[21] Robinson GW, McKnight RA, Smith GH, Hennighausen L (1995). Mammary epithelial cells undergo secretory differentiation in cycling virgins but require pregnancy for the establishment of terminal differentiation.. Development.

[22] Herschkowitz JI, He X, Fan C, Perou CM (2008). The functional loss of the retinoblastoma tumour suppressor is a common event in basal-like and luminal B breast carcinomas.. Breast Cancer Res.

[23] Manie E, Vincent-Salomon A, Lehmann-Che J, Pierron G, Turpin E (2009). High frequency of TP53 mutation in BRCA1 and sporadic basal-like carcinomas but not in BRCA1 luminal breast tumors.. Cancer Res.

[24] Li Z, Tognon CE, Godinho FJ, Yasaitis L, Hock H (2007). ETV6-NTRK3 fusion oncogene initiates breast cancer from committed mammary progenitors via activation of AP1 complex.. Cancer Cell.

[25] Thompson EW, Newgreen DF, Tarin D (2005). Carcinoma invasion and metastasis: a role for epithelial-mesenchymal transition?. Cancer Res.

[26] Zeisberg M, Neilson EG (2009). Biomarkers for epithelial-mesenchymal transitions.. J Clin Invest.

[27] Shamir R, Maron-Katz A, Tanay A, Linhart C, Steinfeld I (2005). EXPANDER–an integrative program suite for microarray data analysis.. BMC Bioinformatics.

[28] Bassuk AG, Barton KP, Anandappa RT, Lu MM, Leiden JM (1998). Expression pattern of the Ets-related transcription factor Elf-1.. Mol Med.

[29] Kendrick H, Regan JL, Magnay FA, Grigoriadis A, Mitsopoulos C (2008). Transcriptome analysis of mammary epithelial subpopulations identifies novel determinants of lineage commitment and cell fate.. BMC Genomics.

[30] Zhang M, Behbod F, Atkinson RL, Landis MD, Kittrell F (2008). Identification of tumor-initiating cells in a p53-null mouse model of breast cancer.. Cancer Res.

[31] Al-Hajj M, Wicha MS, Benito-Hernandez A, Morrison SJ, Clarke MF (2003). Prospective identification of tumorigenic breast cancer cells.. Proc Natl Acad Sci U S A.

[32] Stingl J, Raouf A, Emerman JT, Eaves CJ (2005). Epithelial progenitors in the normal human mammary gland.. J Mammary Gland Biol Neoplasia.

[33] Cho RW, Wang X, Diehn M, Shedden K, Chen GY (2008). Isolation and molecular characterization of cancer stem cells in MMTV-Wnt-1 murine breast tumors.. Stem Cells.

[34] Liu JC, Deng T, Lehal RS, Kim J, Zacksenhaus E (2007). Identification of tumorsphere- and tumor-initiating cells in HER2/Neu-induced mammary tumors.. Cancer Res.

[35] Vassilopoulos A, Wang RH, Petrovas C, Ambrozak D, Koup R (2008). Identification and characterization of cancer initiating cells from BRCA1 related mammary tumors using markers for normal mammary stem cells.. Int J Biol Sci.

[36] Vaillant F, Asselin-Labat ML, Shackleton M, Forrest NC, Lindeman GJ (2008). The mammary progenitor marker CD61/beta3 integrin identifies cancer stem cells in mouse models of mammary tumorigenesis.. Cancer Res.

[37] Grange C, Lanzardo S, Cavallo F, Camussi G, Bussolati B (2008). Sca-1 identifies the tumor-initiating cells in mammary tumors of BALB-neuT transgenic mice.. Neoplasia.

[38] Ansieau S, Bastid J, Doreau A, Morel AP, Bouchet BP (2008). Induction of EMT by twist proteins as a collateral effect of tumor-promoting inactivation of premature senescence.. Cancer Cell.

[39] Strickland P, Shin GC, Plump A, Tessier-Lavigne M, Hinck L (2006). Slit2 and netrin 1 act synergistically as adhesive cues to generate tubular bi-layers during ductal morphogenesis.. Development.

[40] Greenberg JM, Thompson FY, Brooks SK, Shannon JM, Akeson AL (2004). Slit and robo expression in the developing mouse lung.. Dev Dyn.

[41] Erler JT, Bennewith KL, Cox TR, Lang G, Bird D (2009). Hypoxia-induced lysyl oxidase is a critical mediator of bone marrow cell recruitment to form the premetastatic niche.. Cancer Cell.

[42] Ginestier C, Hur MH, Charafe-Jauffret E, Monville F, Dutcher J (2007). ALDH1 is a marker of normal and malignant human mammary stem cells and a predictor of poor clinical outcome.. Cell Stem Cell.

[43] Galang CK, Muller WJ, Foos G, Oshima RG, Hauser CA (2004). Changes in the expression of many Ets family transcription factors and of potential target genes in normal mammary tissue and tumors.. J Biol Chem.

[44] Choi YS, Chakrabarti R, Escamilla-Hernandez R, Sinha S (2009). Elf5 conditional knockout mice reveal its role as a master regulator in mammary alveolar development: failure of Stat5 activation and functional differentiation in the absence of Elf5.. Dev Biol.

[45] Firlej V, Ladam F, Brysbaert G, Dumont P, Fuks F (2008). Reduced tumorigenesis in mouse mammary cancer cells following inhibition of Pea3- or Erm-dependent transcription.. J Cell Sci.

[46] Bloushtain-Qimron N, Yao J, Snyder EL, Shipitsin M, Campbell LL (2008). Cell type-specific DNA methylation patterns in the human breast.. Proc Natl Acad Sci U S A.

[47] Zipori D (2005). The stem state: plasticity is essential, whereas self-renewal and hierarchy are optional.. Stem Cells.

[48] Chang HH, Hemberg M, Barahona M, Ingber DE, Huang S (2008). Transcriptome-wide noise controls lineage choice in mammalian progenitor cells.. Nature.

[49] Kai K, Nagano O, Sugihara E, Arima Y, Sampetrean O (2009). Maintenance of HCT116 colon cancer cell line conforms to a stochastic model but not a cancer stem cell model.. Cancer Sci.

[50] Meyer MJ, Fleming JM, Ali MA, Pesesky MW, Ginsburg E (2009). Dynamic regulation of CD24 and the invasive, CD44posCD24neg phenotype in breast cancer cell lines.. Breast Cancer Res.

[51] Visvader JE (2009). Keeping abreast of the mammary epithelial hierarchy and breast tumorigenesis.. Genes Dev.

[52] Smith GH (2005). Stem cells and mammary cancer in mice.. Stem Cell Rev.

[53] Sun P, Yuan Y, Li A, Li B, Dai X (2010). Cytokeratin expression during mouse embryonic and early postnatal mammary gland development.. Histochem Cell Biol.

[54] Buono KD, Robinson GW, Martin C, Shi S, Stanley P (2006). The canonical Notch/RBP-J signaling pathway controls the balance of cell lineages in mammary epithelium during pregnancy.. Dev Biol.

[55] McCaffrey LM, Macara IG (2009). The Par3/aPKC interaction is essential for end bud remodeling and progenitor differentiation during mammary gland morphogenesis.. Genes Dev.

[56] Stingl J, Eirew P, Ricketson I, Shackleton M, Vaillant F (2006). Purification and unique properties of mammary epithelial stem cells.. Nature.

[57] Nelson CM, Vanduijn MM, Inman JL, Fletcher DA, Bissell MJ (2006). Tissue geometry determines sites of mammary branching morphogenesis in organotypic cultures.. Science.

[58] Gilles C, Polette M, Zahm JM, Tournier JM, Volders L (1999). Vimentin contributes to human mammary epithelial cell migration.. J Cell Sci.

[59] Curtis SJ, Sinkevicius KW, Li D, Lau AN, Roach RR (2010). Primary Tumor Genotype Is an Important Determinant in Identification of Lung Cancer Propagating Cells.. Cell Stem Cell.

[60] Smalley MJ, Clarke RB (2005). The mammary gland “side population”: a putative stem/progenitor cell marker?. J Mammary Gland Biol Neoplasia.

[61] Liu S, Ginestier C, Charafe-Jauffret E, Foco H, Kleer CG (2008). BRCA1 regulates human mammary stem/progenitor cell fate.. Proc Natl Acad Sci U S A.

[62] Dakir EL, Feigenbaum L, Linnoila RI (2008). Constitutive expression of human keratin 14 gene in mouse lung induces premalignant lesions and squamous differentiation.. Carcinogenesis.

[63] Sleeman KE, Kendrick H, Robertson D, Isacke CM, Ashworth A (2007). Dissociation of estrogen receptor expression and in vivo stem cell activity in the mammary gland.. J Cell Biol.

[64] Lennon VA, Unger M, Dulbecco R (1978). Thy-1: a differentiation marker of potential mammary myoepithelial cells in vitro.. Proc Natl Acad Sci U S A.

[65] Rudland PS, Warburton MJ, Monaghan P, Ritter MA (1982). Thy-1 antigen on normal and neoplastic rat mammary tissues: changes in location and amount of antigen during differentiation of cultured stem cells.. J Natl Cancer Inst.

[66] Karnoub AE, Dash AB, Vo AP, Sullivan A, Brooks MW (2007). Mesenchymal stem cells within tumour stroma promote breast cancer metastasis.. Nature.

[67] Reiman JM, Knutson KL, Radisky DC (2010). Immune promotion of epithelial-mesenchymal transition and generation of breast cancer stem cells.. Cancer Res.

[68] Santisteban M, Reiman JM, Asiedu MK, Behrens MD, Nassar A (2009). Immune-induced epithelial to mesenchymal transition in vivo generates breast cancer stem cells.. Cancer Res.

[69] Cannito S, Novo E, Compagnone A, Valfre di Bonzo L, Busletta C (2008). Redox mechanisms switch on hypoxia-dependent epithelial-mesenchymal transition in cancer cells.. Carcinogenesis.

[70] Yang MH, Wu MZ, Chiou SH, Chen PM, Chang SY (2008). Direct regulation of TWIST by HIF-1alpha promotes metastasis.. Nat Cell Biol.

[71] Moen I, Oyan AM, Kalland KH, Tronstad KJ, Akslen LA (2009). Hyperoxic treatment induces mesenchymal-to-epithelial transition in a rat adenocarcinoma model.. PLoS One.

[72] Huang S (2009). Reprogramming cell fates: reconciling rarity with robustness.. Bioessays.

[73] Gumireddy K, Li A, Gimotty PA, Klein-Szanto AJ, Showe LC (2009). KLF17 is a negative regulator of epithelial-mesenchymal transition and metastasis in breast cancer.. Nat Cell Biol.

[74] Yang J, Mani SA, Donaher JL, Ramaswamy S, Itzykson RA (2004). Twist, a master regulator of morphogenesis, plays an essential role in tumor metastasis.. Cell.

[75] Dexter DL, Kowalski HM, Blazar BA, Fligiel Z, Vogel R (1978). Heterogeneity of tumor cells from a single mouse mammary tumor.. Cancer Res.

[76] Mollaaghababa R, Pavan WJ (2003). The importance of having your SOX on: role of SOX10 in the development of neural crest-derived melanocytes and glia.. Oncogene.

[77] Hargreaves DC, Horng T, Medzhitov R (2009). Control of inducible gene expression by signal-dependent transcriptional elongation.. Cell.

[78] Iliopoulos D, Hirsch HA, Struhl K (2009). An epigenetic switch involving NF-kappaB, Lin28, Let-7 MicroRNA, and IL6 links inflammation to cell transformation.. Cell.

[79] Smalley MJ, Titley J, O'Hare MJ (1998). Clonal characterization of mouse mammary luminal epithelial and myoepithelial cells separated by fluorescence-activated cell sorting.. In Vitro Cell Dev Biol Anim.

[80] Tzeng YJ, Zimmermann C, Guhl E, Berg B, Avantaggiati ML (1998). SV40 T/t-antigen induces premature mammary gland involution by apoptosis and selects for p53 missense mutation in mammary tumors.. Oncogene.

