# Supplementary figures and images for: Tumorigenic WAP-T Mouse Mammary Carcinoma Cells: A Model for a Self-Reproducing Homeostatic Cancer Cell System
Source: PLoS One. 2010 Aug 11;5(8):e12103. doi: 10.1371/journal.pone.0012103 (PMC2920333; doi:10.1371/journal.pone.0012103)

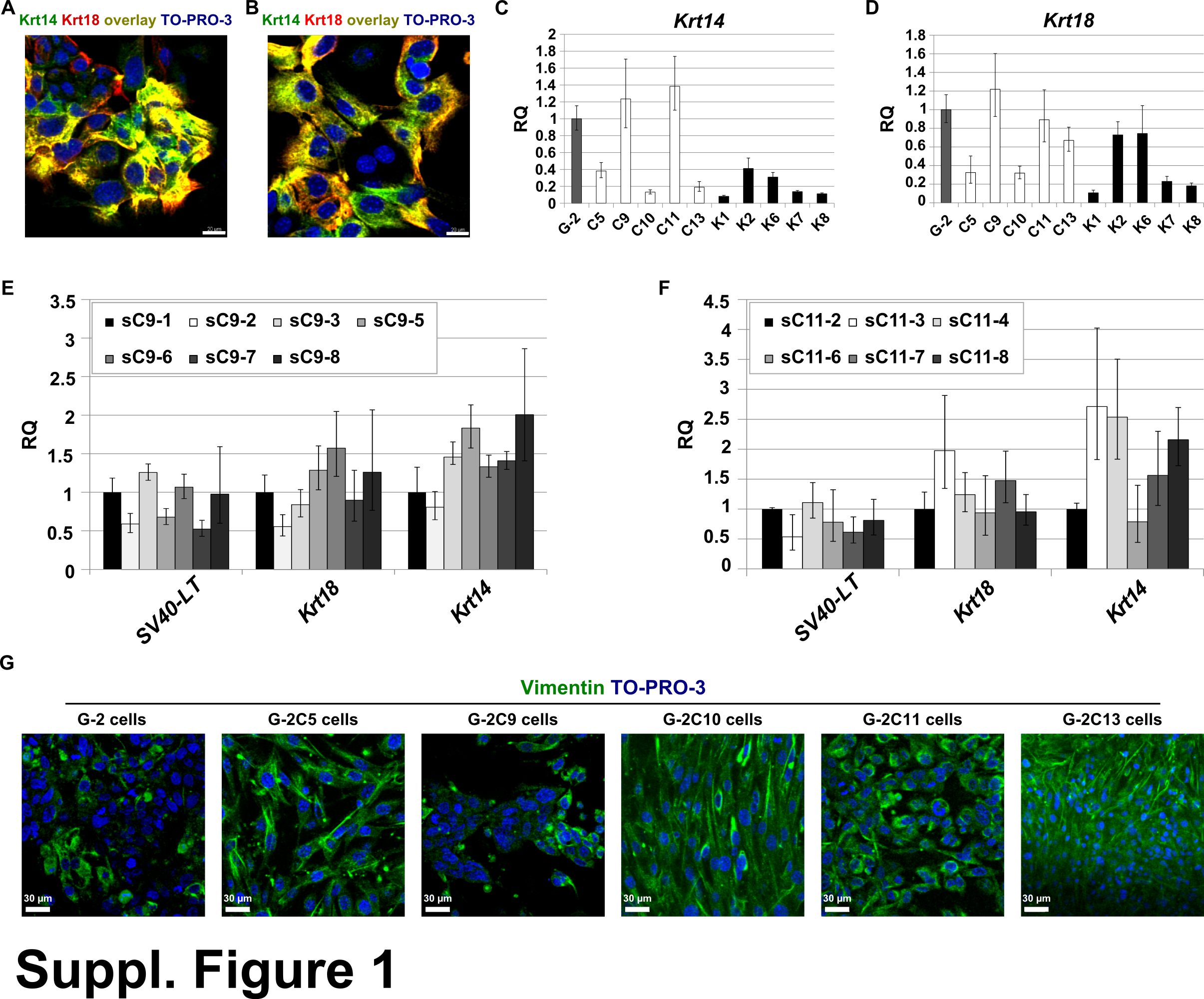

Supplement: Figure S1 — Expression of intermediate filament proteins in cells of G-2 subclones. (A, B) Confocal images of cells of G-2 clones C9 (A) and C11 (B) stained with antibodies against keratin 14 (green) and keratin 18 (red). Nuclei were visualized with TO-PRO-3. Confocal sections were deconvoluted using Huygens Essential and processed with Imaris software. (C, D) 5 clones of the first G-2 cloning (C5, C9, C10, C11, and C13: late passages [P>10]) and 5 of the second cloning (K1, K2, K6, K7, and K8: early passages [P<3]) were subjected to real-time qPCR analysis for Krt14 (C) and Krt18 (D) expression. Gapdh was used as housekeeping gene and the respective results were calibrated on parental G-2 cell expression values. (E, F). 7 and 6 secondary clones, respectively, derived from primary clones G-2C9 (E: sC9-1, sC9-2, sC9-3, sC9-5, sC9-6, sC9-7 and sC9-8) and G-2C11 (F: sC11-2, sC11-3, sC11-4, sC11-6, sC11-7, and sC11-8) were subjected to real-time qPCR analysis for SV40-LT, Krt14 and Krt18 expression. Gapdh was used as housekeeping gene and the respective results were calibrated on sC9-1 and sC11-2 expression values. (G) Confocal images of G-2 cells and subclones G-2C5, G-2C9, G-2C10 and G-2C13 stained for vimentin (green). Nuclei were visualized with TO-PRO-3. Confocal sections were deconvoluted using Huygens Essential and processed with Imaris software. Scale bar: A and B: 20 µm; G: 30 µm. (2.15 MB TIF) [file pone.0012103.s003.tif]

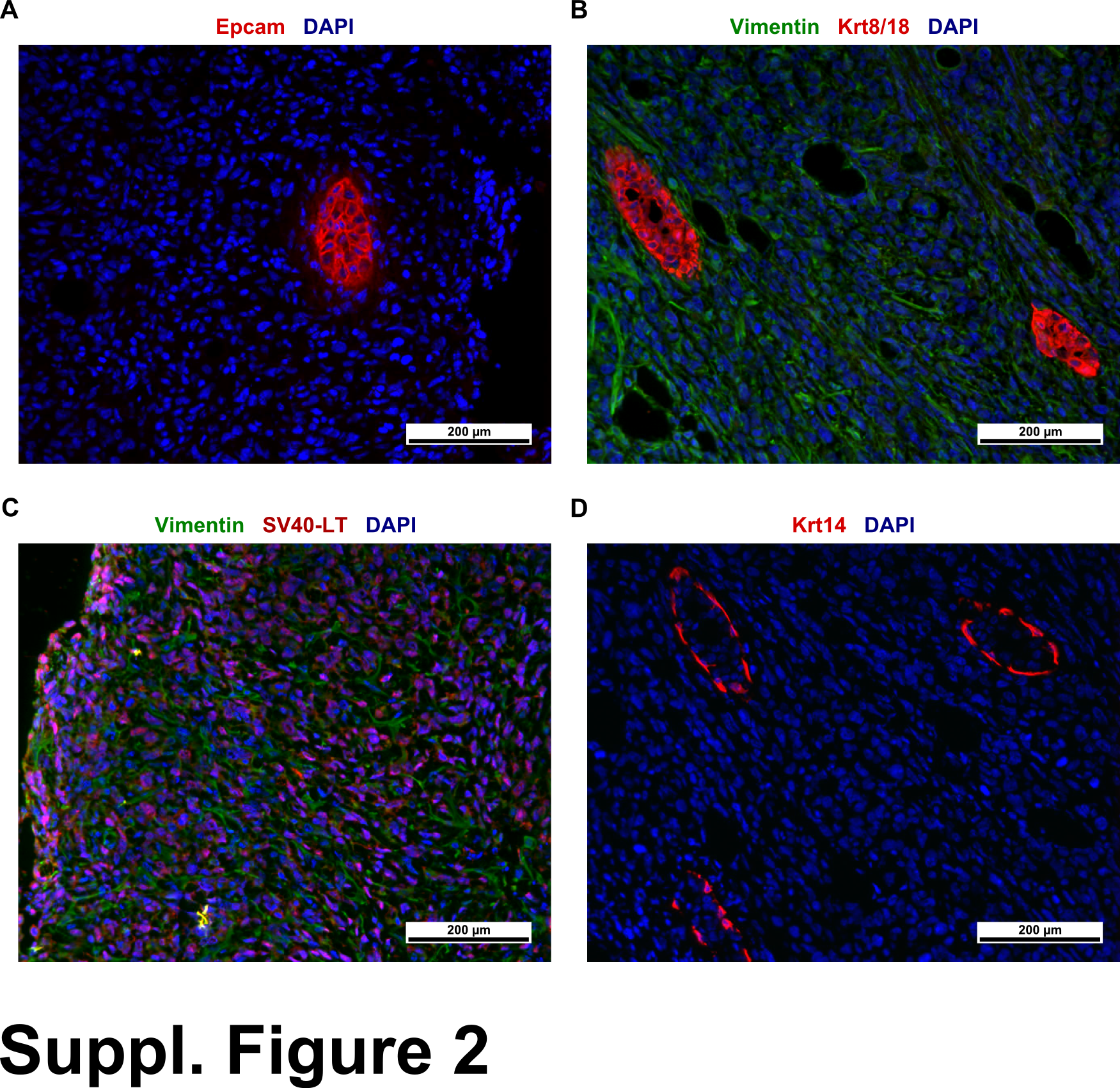

Supplement: Figure S2 — Characterization of transplanted G-2 tumor in BALB/c wild-type recipient mouse. (A–D) Immunostaining of a G-2 tumor in BALB/c recipient mouse for (A) Epcam (red), (B) keratin 8/18 (red) and vimentin (green), (C) SV40-LT (red) and vimentin (green), and (D) keratin 14 (red). Residual structures of the normal mammary gland were observed in A (positive for Epcam), B (positive for keratin 8/18) and C (positive for keratin 14). Nuclei were visualized with DAPI. Scale bars: 200 µm. (4.05 MB TIF) [file pone.0012103.s004.tif]

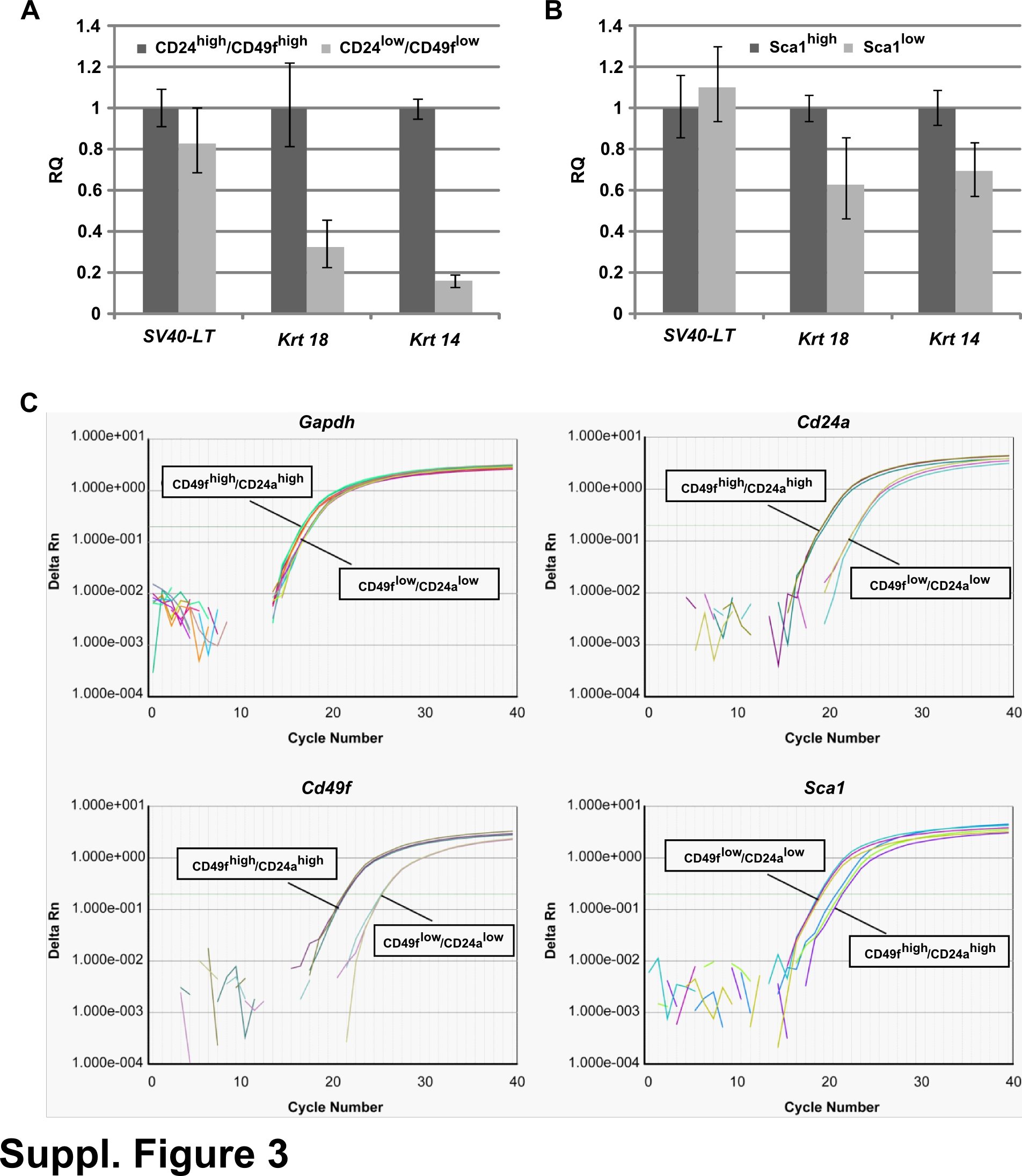

Supplement: Figure S3 — qPCR analysis of G-2 subsets. (A, B) FACS-sorted CD49fhigh/CD24ahigh and CD49flow/CD24alow (A) or Sca1high and Sca1low (B) G-2 subpopulations were subjected to real-time qPCR analysis for SV40-LT, Krt14 and Krt18 expression. Gapdh was used as housekeeping gene and the respective results were calibrated on the expression values of CD49flow/CD24alow and Sca1low subpopulations. (C) Real-time amplification plots of CD49fhigh/CD24ahigh and CD49flow/CD24alow samples for Gapdh, Cd24a, Cd49f, and Sca1 shown on a logarithmic scale Delta Rn. (0.87 MB TIF) [file pone.0012103.s005.tif]

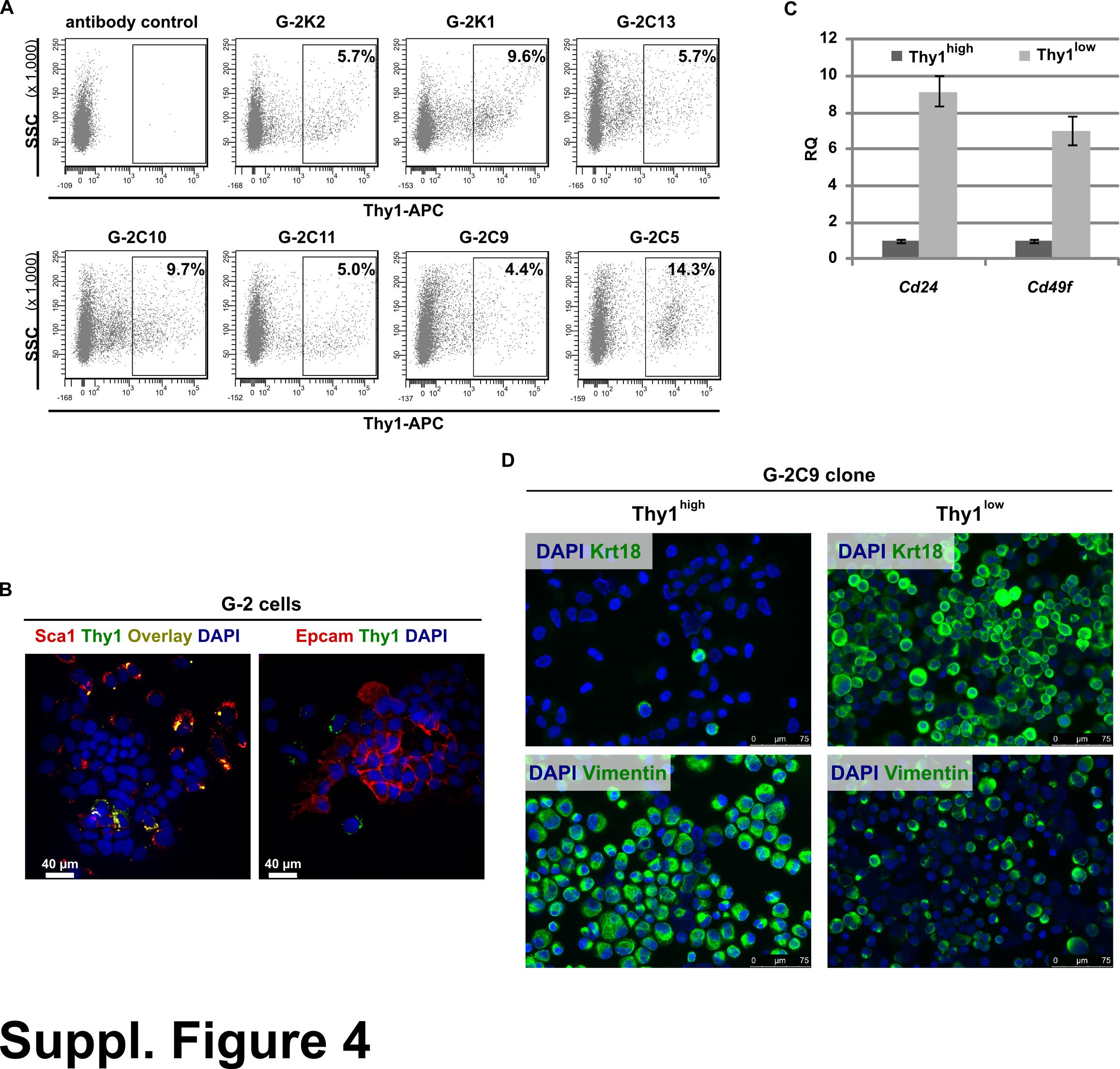

Supplement: Figure S4 — Characterization of the Thy1high cell population of G-2 cells and subclones. (A) Representative FACS dot plots showing the expression of Thy1 in 5 clones of the first G-2 cloning (C5, C9, C10, C11, and C13: late passages [P>10]) and 2 of the second cloning (K1 and K2: early passages [P<3]). The gating was adjusted with the help of an antibody control. (B) Co-immunostaining of G-2 cells grown on coverslips for Sca1 (red) and Thy1 (green). Nuclei were stained with DAPI. (C) Thy1low and Thy1high G-2 subsets were FACS sorted and transcription levels of Cd24a and Cd49f genes were analyzed via real-time qPCR. Gapdh was used as housekeeping gene and results were calibrated on the expression values of the Thy1low subpopulation. (D) Cytospin preparations of FACS-sorted Thy1high and Thy1low G-2C9 cells were stained for keratin 18 (D, upper panels) and vimentin (D, lower panels). Nuclei were stained with DAPI. Scale bar: C: 40 µm; D: 75 µm. (2.37 MB TIF) [file pone.0012103.s006.tif]

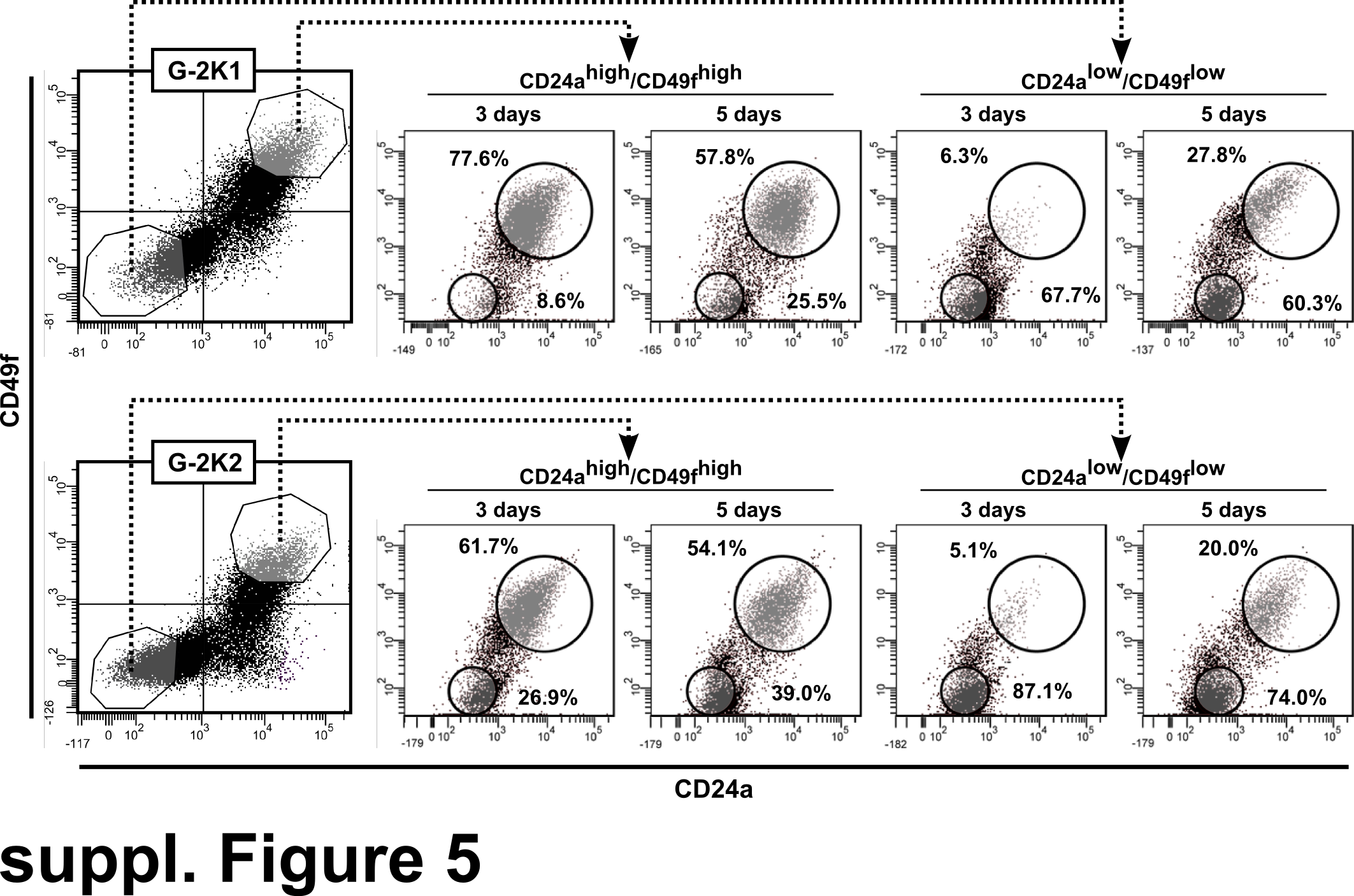

Supplement: Figure S5 — Repopulation activity of G-2K1 and G-2K2 cell subsets. Representative FACS dot plots showing the repopulation activity of two G-2 clones, G-2K1 (upper row) and G-2K2 (lower row), differing in the expression of CD24a and CD49f. CD24ahigh/CD49fhigh and CD24alow/CD49flow subsets were gated during cell sorting to exclude any overlap. 5×10^4^ sorted cells were transferred back into culture and the composition of the culture was analyzed 3 and 5 days later by FACS. (1.04 MB TIF) [file pone.0012103.s007.tif]
